# Self-Normalized Moderate Deviations for Degenerate *U*-Statistics

**DOI:** 10.3390/e27010041

**Published:** 2025-01-07

**Authors:** Lin Ge, Hailin Sang, Qi-Man Shao

**Affiliations:** 1 Division of Arts and Sciences, Mississippi State University at Meridian, Meridian, MS 39307, USA; lge@meridian.msstate.edu; 2 Department of Mathematics, University of Mississippi, University, MS 38677, USA; 3 Department of Statistics and Data Science, Shenzhen International Center for Mathematics, Southern University of Science and Technology, Shenzhen 518055, China; shaoqm@sustech.edu.cn

**Keywords:** moderate deviation, degenerate *U*-statistics, law of the iterated logarithm, self-normalization, 60F15, 60F10, 62E20

## Abstract

In this paper, we study self-normalized moderate deviations for degenerate *U*-statistics of order 2. Let $\left\{X_{i},i\geq 1\right\}$ be i.i.d. random variables and consider symmetric and degenerate kernel functions in the form $h\left(x,y\right)=\sum_{l=1}^{\infty }\lambda_{l}g_{l}\left(x\right)g_{l}\left(y\right)$, where $\lambda_{l}>0$, $Eg_{l}\left(X_{1}\right)=0$, and $g_{l}\left(X_{1}\right)$ is in the domain of attraction of a normal law for all l≥1. Under the condition $\sum_{l=1}^{\infty }\lambda_{l}<\infty$ and some truncated conditions for $\left\{g_{l}\left(X_{1}\right):l\geq 1\right\}$, we show that $\log P\left(\frac{\sum_{1\leq i\neq j\leq n}h\left(X_{i},X_{j}\right)}{\max_{1\leq l<\infty }\lambda_{l}V_{n,l}^{2}}\geq x_{n}^{2}\right)∼-\frac{x_{n}^{2}}{2}$ for $x_{n}\to \infty$ and $x_{n}=o\left(\sqrt{n}\right)$, where $V_{n,l}^{2}=\sum_{i=1}^{n}g_{l}^{2}\left(X_{i}\right)$. As application, a law of the iterated logarithm is also obtained.

## 1. Introduction and Main Results

The recent three decades have witnessed significant developments on self-normalized limit theory, especially on large deviations, Cramér-type moderate deviations, and the law of the iterated logarithm. Compared with the classical limit theorems, these self-normalized limit theorems usually require much less moment assumptions.

Let $X,X_{1},X_{2},\cdots$ be independent identically distributed (i.i.d.) random variables. Set

$$\begin{array}{c} S_{n}=\sum_{i=1}^{n}X_{i}\;\;\mathrm{and}\;\;V_{n}^{2}=\sum_{i=1}^{n}X_{i}^{2}. \end{array}$$

Griffin and Kuelbs [1] obtained a law of the iterated logarithm (LIL) for the self-normalized sum of i.i.d. random variables with distributions in the domain of attraction of a normal or stable law. They proved that

1. If EX=0 and *X* is in the domain of attraction of a normal law, then
  (1) $$\underset{n\to \infty }{lim sup}\frac{S_{n}}{V_{n}{\left(2\log\log n\right)}^{1/2}}=1\;\;a.s.$$
2. If *X* is symmetric and is in the domain of attraction of a stable law, then there exists a positive constant *C* such that
  (2) $$\underset{n\to \infty }{lim sup}\frac{S_{n}}{V_{n}{\left(2\log\log n\right)}^{1/2}}=C\;\;a.s.$$

Shao [2] obtained the following self-normalized moderate deviations and specified the constant *C* in (2). Let $\left\{x_{n},n\geq 1\right\}$ be a sequence of positive numbers such that $x_{n}\to \infty$ and $x_{n}=o\left(\sqrt{n}\right)$ as n→∞.

1. If EX=0 and *X* is in the domain of attraction of a normal law, then
  (3) $$\lim_{n\to \infty }x_{n}^{-2}\log P\left(\frac{S_{n}}{V_{n}}\geq x_{n}\right)=-\frac{1}{2}.$$
2. If *X* is in the domain of attraction of a stable law such that EX=0 with index 1<α<2, or $X_{1}$ is symmetric with index α=1, then
  $$\lim_{n\to \infty }x_{n}^{-2}\log P\left(\frac{S_{n}}{V_{n}}\geq x_{n}\right)=-\beta \left(\alpha ,c_{1},c_{2}\right),$$

where $\beta (\alpha ,c_{1},c_{2})$ is a constant depending on the tail distribution; see [2] for an explicit definition.

Shao [3] refined (3) and obtained the following Cramér-type moderate deviation theorem under a finite third moment: if EX=0 and ${E|X|}^{3}<\infty$, then

(4) $$\frac{P(S_{n}/V_{n}\geq x_{n})}{P(Z\geq x_{n})}\to 1$$

for any $x_{n}\in \left[0,o\left(n^{1/6}\right)\right)$, where *Z* is the standard normal random variable.

Jing, Shao and Wang [4] further extended (4) to general independent random variables under a Lindeberg-type condition, while Shao and Zhou [5] established the result for self-normalized non-linear statistics, which include *U*-statistics as a special case.

The *U*-statistics were introduced by Halmos [6] and Hoeffding [7]. The LIL for nondegenerate *U*-statistics was obtained by Serfling [8]. The LIL for degenerate *U*-statistics was studied by Dehling, Denker and Philipp ([9,10]), Dehling [11], Arcones and Giné [12], Teicher [13], Giné and Zhang [14], and others. Giné, Kwapień, Latała and Zinn [15] provided necessary and sufficient conditions for the LIL of degenerate *U*-statistics of order 2, which was extended to any order by Adamczak and Latała [16].

The main purpose of this paper is to study the self-normalized moderate deviations and the LIL for degenerate *U*-statistics of order 2. Let

$$\begin{array}{c} U_{n}=\frac{1}{n(n-1)}\sum_{1\leq i\neq j\leq n}h\left(X_{i},X_{j}\right), \end{array}$$

where

(5) $$\begin{array}{c} h\left(x,y\right)=\sum_{l=1}^{\infty }\lambda_{l}g_{l}\left(x\right)g_{l}\left(y\right). \end{array}$$

A motivation example for the LIL is the one with the kernel h(x,y)=xy. Obviously, $V_{n}^{2}/\left(2V_{n}^{2}\log\log n\right)\to 0$. Then, via (1) and (2), we have

1. If EX=0 and *X* is in the domain of attraction of a normal law, then
  (6) $$\begin{array}{cc} \; & \underset{n\to \infty }{lim sup}\frac{1}{2V_{n}^{2}\log\log n}|\sum_{1\leq i\neq j\leq n}X_{i}X_{j}|\\ \; & =\underset{n\to \infty }{lim sup}{\left[\frac{S_{n}}{V_{n}{\left(2\log\log n\right)}^{1/2}}\right]}^{2}=1\;\;a.s. \end{array}$$
2. If *X* is symmetric and is in the domain of attraction of a stable law, then there exists a positive constant *C* such that
  $$\begin{array}{cc} \; & \underset{n\to \infty }{lim sup}\frac{1}{2V_{n}^{2}\log\log n}\left|\sum_{1\leq i\neq j\leq n}X_{i}X_{j}\right|\\ \; & =\underset{n\to \infty }{lim sup}{\left[\frac{S_{n}}{V_{n}{\left(2\log\log n\right)}^{1/2}}\right]}^{2}=C^{2}\;\;a.s. \end{array}$$

For the general degenerate kernel *h* defined in (5), we have

$$\begin{array}{ccc} n\left(n-1\right)U_{n} & = & \sum_{l=1}^{\infty }\lambda_{l}\sum_{1\leq i\neq j\leq n}g_{l}\left(X_{i}\right)g_{l}\left(X_{j}\right)\\ & = & \sum_{l=1}^{\infty }\lambda_{l}\left({\left(\sum_{i=1}^{n}g_{l}\left(X_{i}\right)\right)}^{2}-\sum_{i=1}^{n}g_{l}^{2}\left(X_{i}\right)\right). \end{array}$$

Suppose that $g_{l}\left(X\right)$ is in the domain of attraction of a normal law for every l≥1. Then, $L_{l}\left(x\right):=Eg_{l}^{2}\left(X_{1}\right)I(|g_{l}\left(X_{1}\right)\left|\leq x\right)$ is a slowly varying function for all l≥1 as x→∞. Let $\left\{x_{n},n\geq 1\right\}$ be a sequence of positive numbers such that $x_{n}\to \infty$ and $x_{n}=o\left(\sqrt{n}\right)$ as n→∞. For each l≥1, set

(7) $$\begin{array}{ccc} b_{l} & = & \inf\left\{x\geq 1:L_{l}\left(x\right)>0\right\},\\ z_{n,l} & = & \inf\left\{s:s\geq b_{l}+1,\frac{L_{l}\left(s\right)}{s^{2}}\leq \frac{x_{n}^{2}}{n}\right\}. \end{array}$$

Write

$$\begin{array}{c} W_{n}=\frac{n\left(n-1\right)U_{n}}{\max_{1\leq l<\infty }\lambda_{l}\sum_{i=1}^{n}g_{l}^{2}\left(X_{i}\right)}. \end{array}$$

We have the following self-normalized moderate deviation:

**Theorem** **1.** *Let $Eg_{l}\left(X\right)=0$ and $\lambda_{l}\geq 0$ for every l≥1.*

(8) $$\begin{array}{c} \underset{x\in R}{\sup}\frac{\sum_{l=m+1}^{\infty }\lambda_{l}g_{l}^{2}\left(x\right)}{\sum_{1\leq l<\infty }\lambda_{l}g_{l}^{2}\left(x\right)}\to 0\;\;as\;\;m\to \infty \end{array}$$

*and*

(9) $$\begin{array}{c} \lim_{n\to \infty }\frac{Eg_{l}\left(X\right)I(|g_{l}\left(X\right)|\leq z_{n,l})g_{k}\left(X\right)I(|g_{k}\left(X\right)\left|\leq z_{n,k}\right)}{\sqrt{L_{l}\left(z_{n,l}\right)L_{k}\left(z_{n,k}\right)}}\to 0 \end{array}$$

*for any l≠k. Then, for $x_{n}\to \infty$ and $x_{n}=o\left(\sqrt{n}\right)$,*

$$\begin{array}{c} \lim_{n\to \infty }x_{n}^{-2}\log P\left(W_{n}\geq x_{n}^{2}\right)=-\frac{1}{2}. \end{array}$$

As an application, we have the following self-normalized LIL:

**Theorem** **2.** *Under the assumptions in Theorem 1, and instead of (8), we assume that for each l∈[1,∞), there is a constant $c_{l}>0$ such that*

(10) $$\begin{array}{c} \underset{x\in R}{\sup}\frac{\lambda_{l}g_{l}^{2}\left(x\right)}{\sum_{l=1}^{\infty }\lambda_{l}g_{l}^{2}\left(x\right)}\leq c_{l}\;\;and\;\;\sum_{l=1}^{\infty }c_{l}<\infty . \end{array}$$

*Then,*

(11) $$\begin{array}{c} \underset{n\to \infty }{lim sup}\frac{W_{n}}{\log\log n}=2\;\;a.s. \end{array}$$

**Remark** **1.** *We use an example to show that (9) cannot be removed. Let $g_{1}\left(x\right)=x$, $g_{2}\left(x\right)=x^{3}$ and $\lambda_{1}=\lambda_{2}=1$, $\lambda_{l}=0$ for l≥3. Let X be a Rademacher random variable. Then, $n\left(n-1\right)U_{n}=\sum_{1\leq i\neq j\leq n}\left(X_{i}X_{j}+X_{i}^{3}X_{j}^{3}\right)=2\sum_{1\leq i\neq j\leq n}X_{i}X_{j}$ and $W_{n}=2\sum_{1\leq i\neq j\leq n}X_{i}X_{j}/\sum_{i=1}^{n}X_{i}^{2}$. By (6), ${lim sup}_{n\to \infty }W_{n}/\log\log n=4$ a.s. which contradicts (11).*

## 2. Proofs

In the proofs of theorems, we will use the following properties for the slowly varying functions $g_{l}$ (e.g., Bingham et al. [17]). As x→∞,

(12) $$\begin{array}{c} P(|g_{l}\left(X\right)\left|\geq x\right)=o\left(L_{l}\left(x\right)/x^{2}\right), \end{array}$$

(13) $$\begin{array}{c} E|g_{l}\left(X\right)|I(|g_{l}\left(X\right)\left|\geq x\right)=o\left(L_{l}\left(x\right)/x\right), \end{array}$$

(14) $$\begin{array}{c} E|g_{l}{\left(X\right)|}^{p}I(|g_{l}\left(X\right)\left|\leq x\right)=o\left(x^{p-2}L_{l}\left(x\right)\right),\;\;p>2. \end{array}$$

Since $L_{l}\left(s\right)/s^{2}\to 0$ as s→∞ and $L_{l}\left(x\right)$ is right continuous, in (7), $z_{n,l}\to \infty$ and for all sufficiently large *n* values, we have

(15) $$\begin{array}{c} nL_{l}\left(z_{n,l}\right)=x_{n}^{2}z_{n,l}^{2}. \end{array}$$

### 2.1. The Upper Bound of Theorem 1

For each l≥1 and i≥1, denote the truncated function

$$\begin{array}{c} {\bar{g}}_{l}\left(X_{i}\right)=g_{l}\left(X_{i}\right)I\left(|g_{l}\left(X_{i}\right)|\leq z_{n,l}\right). \end{array}$$

Since $Eg_{l}\left(X_{i}\right)=0$, we have

(16) $$\begin{array}{c} E{\bar{g}}_{l}\left(X_{i}\right)=o\left(L_{l}\left(z_{n,l}\right)/z_{n,l}\right)=o\left(x_{n}\sqrt{L_{l}(z_{n,l}}\right)/\sqrt{n}) \end{array}$$

by (13) and (15). For each l≥1, write

$$\begin{array}{c} Y_{n,l}=\sum_{i=1}^{n}g_{l}\left(X_{i}\right),\;\;{\bar{Y}}_{n,l}=\sum_{i=1}^{n}{\bar{g}}_{l}\left(X_{i}\right)\;\;\mathrm{and}\;\;V_{n,l}^{2}=\sum_{i=1}^{n}g_{l}^{2}\left(X_{i}\right). \end{array}$$

By Condition (8), for each 0<ε<1, there exists 1≤m<∞ such that

(17) $$\begin{array}{c} m\max_{1\leq l<\infty }\lambda_{l}V_{n,l}^{2}\geq \sum_{l=1}^{m}\lambda_{l}V_{n,l}^{2}\geq \left(1-\varepsilon \right)\sum_{l=1}^{\infty }\lambda_{l}V_{n,l}^{2}. \end{array}$$

Hence, for $x_{n}\to \infty$,

(18) $$\begin{array}{c} P\left(W_{n}\geq \left(1+\varepsilon \right)x_{n}^{2}\right)\leq P\left(\frac{\sum_{l=1}^{\infty }\lambda_{l}Y_{n,l}^{2}}{\max_{1\leq l<\infty }\lambda_{l}V_{n,l}^{2}}\geq x_{n}^{2}\right). \end{array}$$

Observe that for any random variables ${\left\{U_{l}\right\}}_{l=1}^{\infty }$ and ${\left\{Z_{l}\right\}}_{l=1}^{\infty }$ and any constants x>0 and 0<a<1/3, we have the following via the Cauchy inequality:

(19) $$\begin{array}{ccc} & & P\left(\sum_{l}{\left(U_{l}+Z_{l}\right)}^{2}\geq x\right)\\ & \leq & P\left(\sum_{l}U_{l}^{2}\geq {\left(1-a\right)}^{2}x\right)+P\left(\sum_{l}Z_{l}^{2}\geq a^{2}x\right)\\ & \leq & P\left(\sum_{l}U_{l}^{2}\geq \left(1-2a\right)x\right)+P\left(\sum_{l}Z_{l}^{2}\geq a^{2}x\right). \end{array}$$

For any 0<ε<1/3, by (18) and (19), we have

(20) $$\begin{array}{ccc} & & P\left(W_{n}\geq \left(1+\varepsilon \right)x_{n}^{2}\right)\\ & \leq & P\left(\frac{\sum_{l=1}^{\infty }\lambda_{l}{\left(\sum_{i=1}^{n}g_{l}\left(X_{i}\right)I\left(|g_{l}\left(X_{i}\right)|>z_{n,l}\right)\right)}^{2}}{\max_{1\leq l<\infty }\lambda_{l}V_{n,l}^{2}}\geq \varepsilon^{2}x_{n}^{2}\right)\\ & & +P\left(\frac{\sum_{l=1}^{\infty }\lambda_{l}{\bar{Y}}_{n,l}^{2}}{\max_{1\leq l<\infty }\lambda_{l}V_{n,l}^{2}}\geq \left(1-2\varepsilon \right)x_{n}^{2}\right). \end{array}$$

For any integer m≥1 and any constant $C_{1}>0$ with $C_{1}\varepsilon <1$, we have

(21) $$\begin{array}{ccc} & & P\left(\frac{\sum_{l=1}^{\infty }\lambda_{l}{\bar{Y}}_{n,l}^{2}}{\max_{1\leq l<\infty }\lambda_{l}V_{n,l}^{2}}\geq \left(1-2\varepsilon \right)x_{n}^{2}\right)\\ & \leq & P\left(\max_{1\leq l<\infty }\lambda_{l}V_{n,l}^{2}\leq \frac{n}{\varepsilon }\sum_{l=m+1}^{\infty }\lambda_{l}L_{l}\left(z_{n,l}\right)\right)\\ & & +P\left(\max_{1\leq l<\infty }\lambda_{l}V_{n,l}^{2}\leq \left(1-\varepsilon \right)n\max_{1\leq l\leq m}\lambda_{l}L_{l}\left(z_{n,l}\right)\right)\\ & & +P\left(\frac{\sum_{l=m+1}^{\infty }\lambda_{l}{\bar{Y}}_{n,l}^{2}}{\left(n/\varepsilon \right)\sum_{l=m+1}^{\infty }\lambda_{l}L_{l}\left(z_{n,l}\right)}\geq C_{1}\varepsilon \left(1-2\varepsilon \right)x_{n}^{2}\right)\\ & & +P\left(\frac{\sum_{l=1}^{m}\lambda_{l}{\bar{Y}}_{n,l}^{2}}{\left(1-\varepsilon \right)n\max_{1\leq l\leq m}\lambda_{l}L_{l}\left(z_{n,l}\right)}\geq \left(1-C_{1}\varepsilon \right)\left(1-2\varepsilon \right)x_{n}^{2}\right). \end{array}$$

Applying (21) to (20), we have

(22) $$\begin{array}{ccc} & & P\left(W_{n}\geq \left(1+\varepsilon \right)x_{n}^{2}\right)\\ & \leq & P\left(\max_{1\leq l<\infty }\lambda_{l}V_{n,l}^{2}\leq \frac{n}{\varepsilon }\sum_{l=m+1}^{\infty }\lambda_{l}L_{l}\left(z_{n,l}\right)\right)\\ & & +P\left(\max_{1\leq l<\infty }\lambda_{l}V_{n,l}^{2}\leq \left(1-\varepsilon \right)n\max_{1\leq l\leq m}\lambda_{l}L_{l}\left(z_{n,l}\right)\right)\\ & & +P\left(\frac{\sum_{l=1}^{\infty }\lambda_{l}{\left(\sum_{i=1}^{n}g_{l}\left(X_{i}\right)I\left(|g_{l}\left(X_{i}\right)|>z_{n,l}\right)\right)}^{2}}{\max_{1\leq l<\infty }\lambda_{l}V_{n,l}^{2}}\geq \varepsilon^{2}x_{n}^{2}\right)\\ & & +P\left(\frac{\sum_{l=m+1}^{\infty }\lambda_{l}{\bar{Y}}_{n,l}^{2}}{n\sum_{l=m+1}^{\infty }\lambda_{l}L_{l}\left(z_{n,l}\right)}\geq C_{1}\left(1-2\varepsilon \right)x_{n}^{2}\right)\\ & & +P\left(\frac{\sum_{l=1}^{m}\lambda_{l}{\bar{Y}}_{n,l}^{2}}{n\max_{1\leq l\leq m}\lambda_{l}L_{l}\left(z_{n,l}\right)}\geq \left(1-C_{1}\varepsilon \right){\left(1-2\varepsilon \right)}^{2}x_{n}^{2}\right)\\ & := & I_{1,1}+I_{1,2}+I_{2}+I_{3}+I_{4}. \end{array}$$

### 2.2. Estimation of $I_{1,1}$ and $I_{1,2}$

**Proposition** **1.** *For m≥1 that is sufficiently large,*

(23) $$\begin{array}{c} I_{1,1}=P\left(\max_{1\leq l<\infty }\lambda_{l}V_{n,l}^{2}\leq \frac{n}{\varepsilon }\sum_{l=m+1}^{\infty }\lambda_{l}L_{l}\left(z_{n,l}\right)\right)\leq \exp\left(-2x_{n}^{2}\right) \end{array}$$

*and for any constants δ>0 and 0<η<1,*

(24) $$\begin{array}{c} P\left(2m\max_{1\leq l<\infty }\lambda_{l}V_{n,l}^{2}\leq \left(1-\eta \right)n\sum_{1\leq l<\infty }\lambda_{l}L_{l}\left(z_{n,l}\right)\right)\leq \exp\left(-2x_{n}^{2}\right), \end{array}$$

(25) $$\begin{array}{ccc} & & P\left(\max_{1\leq l<\infty }\lambda_{l}\sum_{i=1}^{n}g_{l}^{2}\left(X_{i}\right)I(|g_{l}\left(X_{i}\right)\left|\leq \delta z_{n,l}\right)\leq \left(1-\eta \right)n\max_{1\leq l<\infty }\lambda_{l}L_{l}\left(z_{n,l}\right)\right)\\ & \leq & \exp(-2x_{n}^{2}). \end{array}$$

*In particular,*

$$\begin{array}{c} I_{1,2}\leq P\left(\max_{1\leq l<\infty }\lambda_{l}V_{n,l}^{2}\leq \left(1-\varepsilon \right)n\max_{1\leq l<\infty }\lambda_{l}L_{l}\left(z_{n,l}\right)\right)\leq \exp\left(-2x_{n}^{2}\right). \end{array}$$

**Proof.**  We shall apply the following exponential inequality (see, e.g., Theorem 2.19 of de la Peña, Lai and Shao [18]). If $Y_{1},\ldots ,Y_{n}$ are independent random variables with $Y_{i}\geq 0$, $\mu_{n}=\sum_{i=1}^{n}EY_{i}$ and $B_{n}^{2}=\sum_{i=1}^{n}EY_{i}^{2}<\infty$, then for $0<x<\mu_{n}$,

$$\begin{array}{c} P\left(\sum_{i=1}^{n}Y_{i}\leq x\right)\leq \exp\left(-\frac{{\left(\mu_{n}-x\right)}^{2}}{2B_{n}^{2}}\right). \end{array}$$

By (8), $\sum_{l=m+1}^{\infty }\lambda_{l}V_{n,l}^{2}/\sum_{l=1}^{\infty }\lambda_{l}V_{n,l}^{2}\to 0$ as m→∞. Then, by (17),

(26) $$\begin{array}{c} \frac{\sum_{l=m+1}^{\infty }\lambda_{l}V_{n,l}^{2}}{\max_{1\leq l<\infty }\lambda_{l}V_{n,l}^{2}}\to 0\;\;\;as\;\;m\to \;\infty . \end{array}$$

Hence,

$$\begin{array}{c} \varepsilon \max_{1\leq l<\infty }\lambda_{l}V_{n,l}^{2}\geq 2\sum_{l=m+1}^{\infty }\lambda_{l}V_{n,l}^{2}\geq 2\sum_{l=m+1}^{\infty }\lambda_{l}\sum_{i=1}^{n}{\bar{g}}_{l}^{2}\left(X_{i}\right). \end{array}$$

Then,

(27) $$\begin{array}{ccc} I_{1,1} & \leq & P\left(\sum_{l=m+1}^{\infty }\lambda_{l}\sum_{i=1}^{n}{\bar{g}}_{l}^{2}\left(X_{i}\right)\leq \frac{n}{2}\sum_{l=m+1}^{\infty }\lambda_{l}L_{l}\left(z_{n,l}\right)\right)\\ & \leq & \exp\left(-\frac{{\left(n\sum_{l=m+1}^{\infty }\lambda_{l}L_{l}\left(z_{n,l}\right)/2\right)}^{2}}{2nE{\left(\sum_{l=m+1}^{\infty }\lambda_{l}{\bar{g}}_{l}^{2}\left(X_{1}\right)\right)}^{2}}\right). \end{array}$$

By Minkowski’s integral inequality, (14) and (15),

(28) $$\begin{array}{ccc} E{\left(\sum_{l=m+1}^{\infty }\lambda_{l}{\bar{g}}_{l}^{2}\left(X_{1}\right)\right)}^{2} & \leq & {\left\{\sum_{l=m+1}^{\infty }\lambda_{l}{\left(E{\bar{g}}_{l}^{4}\left(X_{1}\right)\right)}^{1/2}\right\}}^{2}\\ & = & o{\left(\frac{\sqrt{n}}{x_{n}}\sum_{l=m+1}^{\infty }\lambda_{l}L_{l}\left(z_{n,l}\right)\right)}^{2}. \end{array}$$

Therefore, (23) follows from (27) and (28). To show (24), notice that by (17),

$$\begin{array}{c} m\max_{1\leq l<\infty }\lambda_{l}V_{n,l}^{2}\geq \left(1-\eta \right)\sum_{l=1}^{\infty }\lambda_{l}V_{n,l}^{2}\geq \left(1-\eta \right)\sum_{l=1}^{\infty }\lambda_{l}\sum_{i=1}^{n}{\bar{g}}_{l}^{2}\left(X_{i}\right). \end{array}$$

Then,

$$\begin{array}{ccc} & & P\left(2m\max_{1\leq l<\infty }\lambda_{l}V_{n,l}^{2}\leq \left(1-\eta \right)n\sum_{1\leq l<\infty }\lambda_{l}L_{l}\left(z_{n,l}\right)\right)\\ & \leq & P\left(\sum_{l=1}^{\infty }\lambda_{l}\sum_{i=1}^{n}{\bar{g}}_{l}^{2}\left(X_{i}\right)\leq \frac{n}{2}\sum_{l=1}^{\infty }\lambda_{l}L_{l}\left(z_{n,l}\right)\right)\\ & \leq & \exp\left(-\frac{({\left(\frac{n}{2}\sum_{l=1}^{\infty }\lambda_{l}L_{l}\left(z_{n,l}\right)\right)}^{2}}{2nE{\left(\sum_{l=1}^{\infty }\lambda_{l}{\bar{g}}_{l}^{2}\left(X_{1}\right)\right)}^{2}}\right). \end{array}$$

Similar to the proof of $I_{1,1}$ as in (27) and (28), we have (24).

To show (25), let

$$\begin{array}{c} l_{n}=\min\left\{l:\lambda_{l}L_{l}\left(z_{n,l}\right)=\max_{1\leq l<\infty }\lambda_{l}L_{l}\left(z_{n,l}\right)\right\}. \end{array}$$

Then,

$$\begin{array}{ccc} & & P\left(\max_{1\leq l<\infty }\lambda_{l}\sum_{i=1}^{n}g_{l}^{2}\left(X_{i}\right)I(|g_{l}\left(X_{i}\right)\left|\leq \delta z_{n,l}\right)\leq \left(1-\eta \right)n\max_{1\leq l<\infty }\lambda_{l}L_{l}\left(z_{n,l}\right)\right)\\ & \leq & P\left(\sum_{i=1}^{n}\lambda_{l_{n}}g_{l_{n}}^{2}\left(X_{i}\right)I(|g_{l_{n}}\left(X_{i}\right)\left|\leq \delta z_{n,l_{n}}\right)\leq \left(1-\eta \right)n\lambda_{l_{n}}L_{l_{n}}\left(z_{n,l_{n}}\right)\right)\\ & \leq & \exp\left(-\frac{{\left(1-\left(1-\eta \right)\right)}^{2}{\left(n\lambda_{l_{n}}L_{l_{n}}\left(z_{n,l_{n}}\right)\right)}^{2}}{2n\lambda_{l_{n}}^{2}Eg_{l_{n}}^{4}\left(X_{1}\right)I(|g_{l_{n}}\left(X_{1}\right)\left|\leq \delta z_{n,l_{n}}\right)}\right)\\ & = & \exp\left(-\frac{\eta^{2}{\left(n\lambda_{l_{n}}L_{l_{n}}\left(z_{n,l_{n}}\right)\right)}^{2}}{2n\lambda_{l_{n}}^{2}o\left(nL_{l_{n}}^{2}\left(\delta z_{n,l_{n}}\right)/x_{n}^{2}\right)}\right). \end{array}$$

Since $L_{l}\left(\delta z_{n,l}\right)/L_{l}\left(z_{n,l}\right)\to 1$, (25) follows. □

### 2.3. Estimation of $I_{2}$

**Proposition** **2.**
$$\begin{array}{c} I_{2}=P\left(\frac{\sum_{l=1}^{\infty }\lambda_{l}{\left\{\sum_{i=1}^{n}g_{l}\left(X_{i}\right)I\left(|g_{l}\left(X_{i}\right)|>z_{n,l}\right)\right\}}^{2}}{\max_{1\leq l<\infty }\lambda_{l}V_{n,l}^{2}}\geq \varepsilon^{2}x_{n}^{2}\right)\leq \exp\left(-2x_{n}^{2}\right). \end{array}$$

**Proof.**  Via the Cauchy–Schwarz inequality,

$$\begin{array}{ccc} & & \frac{\sum_{l=1}^{\infty }\lambda_{l}{\left\{\sum_{i=1}^{n}\left|g_{l}\left(X_{i}\right)\right|I\left(|g_{l}\left(X_{i}\right)|>z_{n,l}\right)\right\}}^{2}}{\max_{1\leq l<\infty }\lambda_{l}V_{n,l}^{2}}\\ & \leq & \frac{\sum_{l=1}^{\infty }\lambda_{l}\sum_{i=1}^{n}g_{l}^{2}\left(X_{i}\right)\sum_{i=1}^{n}I\left(|g_{l}\left(X_{i}\right)|>z_{n,l}\right)}{\max_{1\leq l<\infty }\lambda_{l}V_{n,l}^{2}}. \end{array}$$

By (17), the sum of the diagonal terms is as follows:

$$\begin{array}{c} \frac{\sum_{l=1}^{\infty }\lambda_{l}\sum_{i=1}^{n}g_{l}^{2}\left(X_{i}\right)I\left(|g_{l}\left(X_{i}\right)|>z_{n,l}\right)}{\max_{1\leq l<\infty }\lambda_{l}V_{n,l}^{2}}\leq \frac{\varepsilon^{2}x_{n}^{2}}{2}. \end{array}$$

(29) $$\begin{array}{ccc} I_{2} & \leq & P\left(\frac{\sum_{1\leq i\neq j\leq n}\sum_{l=1}^{\infty }\lambda_{l}g_{l}^{2}\left(X_{i}\right)I(|g_{l}\left(X_{j}\right)\left|>z_{n,l}\right)}{\max_{1\leq l<\infty }\lambda_{l}\sum_{i=1}^{n}g_{l}^{2}\left(X_{i}\right)}\geq \frac{\varepsilon^{2}x_{n}^{2}}{2}\right)\\ & \leq & P\left(\frac{\sum_{1\leq i<j\leq n}\sum_{l=1}^{\infty }\lambda_{l}g_{l}^{2}\left(X_{i}\right)I(|g_{l}\left(X_{j}\right)\left|>z_{n,l}\right)}{\max_{1\leq l<\infty }\lambda_{l}\sum_{i=1}^{n}g_{l}^{2}\left(X_{i}\right)}\geq \frac{\varepsilon^{2}x_{n}^{2}}{4}\right)\\ & + & P\left(\frac{\sum_{1\leq j<i\leq n}\sum_{l=1}^{\infty }\lambda_{l}g_{l}^{2}\left(X_{i}\right)I(|g_{l}\left(X_{j}\right)\left|>z_{n,l}\right)}{\max_{1\leq l<\infty }\lambda_{l}\sum_{i=1}^{n}g_{l}^{2}\left(X_{i}\right)}\geq \frac{\varepsilon^{2}x_{n}^{2}}{4}\right)\\ & \leq & P\left(\sum_{2\leq j\leq n}\frac{\sum_{1\leq i<j}\sum_{l=1}^{\infty }\lambda_{l}g_{l}^{2}\left(X_{i}\right)I(|g_{l}\left(X_{j}\right)\left|>z_{n,l}\right)}{\max_{1\leq l<\infty }\lambda_{l}\sum_{1\leq i<j}g_{l}^{2}\left(X_{i}\right)}\geq \frac{\varepsilon^{2}x_{n}^{2}}{4}\right)\\ & + & P\left(\sum_{1\leq j<n}\frac{\sum_{j<i\leq n}\sum_{l=1}^{\infty }\lambda_{l}g_{l}^{2}\left(X_{i}\right)I(|g_{l}\left(X_{j}\right)\left|>z_{n,l}\right)}{\max_{1\leq l<\infty }\lambda_{l}\sum_{j<i\leq n}g_{l}^{2}\left(X_{i}\right)}\geq \frac{\varepsilon^{2}x_{n}^{2}}{4}\right)\\ & = & I_{2,1}+I_{2,2}. \end{array}$$

Let

$$\begin{array}{c} \phi_{j}=\frac{\sum_{1\leq i<j}\sum_{l=1}^{\infty }\lambda_{l}g_{l}^{2}\left(X_{i}\right)I(|g_{l}\left(X_{j}\right)\left|>z_{n,l}\right)}{\max_{1\leq l<\infty }\lambda_{l}\sum_{1\leq i<j}g_{l}^{2}\left(X_{i}\right)}. \end{array}$$

Then, for any constant t>0,

(30) $$\begin{array}{c} I_{2,1}\leq Ee^{t\sum_{j=2}^{n}\phi_{j}}e^{-t\varepsilon^{2}x_{n}^{2}/4}. \end{array}$$

Let $E_{j}$ be the expectation of $X_{j}$ for 2≤j≤n. Then,

(31) $$\begin{array}{c} Ee^{t\sum_{j=2}^{n}\phi_{j}}=E\left(e^{t\sum_{j=2}^{n-1}\phi_{j}}E_{n}e^{t\phi_{n}}\right). \end{array}$$

Since $|e^{s}-1|\leq e^{0\vee s}\left|s\right|$ for any s∈R and $0\leq \phi_{n}\leq m$ for some sufficiently large *m* value, then

$$\begin{array}{ccc} \left|E_{n}e^{t\phi_{n}}-1\right| & \leq & e^{mt}tE_{n}\phi_{n}\\ & = & \frac{e^{mt}t\sum_{1\leq i<n}\sum_{l=1}^{\infty }\lambda_{l}g_{l}^{2}\left(X_{i}\right)P(|g_{l}\left(X_{n}\right)\left|>z_{n,l}\right)}{\max_{1\leq l<\infty }\lambda_{l}\sum_{1\leq i<n}g_{l}^{2}\left(X_{i}\right)}. \end{array}$$

By (12) and (15), we have $P(|g_{l}\left(X_{n}\right)\left|>z_{n,l}\right)=o\left(x_{n}^{2}/n\right)$. Then, together with (17),

(32) $$\begin{array}{c} E_{n}e^{t\phi_{n}}=1+o\left(x_{n}^{2}/n\right)=e^{o(x_{n}^{2}/n)}. \end{array}$$

Applying (32) to (31), we have

$$\begin{array}{c} Ee^{t\sum_{j=2}^{n}\phi_{j}}=e^{o(x_{n}^{2}/n)}Ee^{t\sum_{j=2}^{n-1}\phi_{j}}. \end{array}$$

Similarly,

$$\begin{array}{ccc} Ee^{t\sum_{j=2}^{n-1}\phi_{j}} & = & E(e^{t\sum_{j=2}^{n-2}\phi_{j}}E_{n-1}e^{t\phi_{n-1}})\\ & = & e^{o(x_{n}^{2}/n)}Ee^{t\sum_{j=2}^{n-2}\phi_{j}}. \end{array}$$

Continue this process from $X_{n}$ to $X_{1}$. We conclude that

(33) $$\begin{array}{c} Ee^{t\sum_{j=2}^{n}\phi_{j}}=e^{n\times o(x_{n}^{2}/n)}=e^{o(x_{n}^{2})}. \end{array}$$

Applying (33) to (30) and letting $t=16/\varepsilon^{2}$, we have

(34) $$\begin{array}{c} I_{2,1}\leq \exp\left(-3x_{n}^{2}\right). \end{array}$$

By the same argument,

(35) $$\begin{array}{c} I_{2,2}\leq \exp\left(-3x_{n}^{2}\right). \end{array}$$

Combining (29), (34) and (35), we obtain the proposition. □

### 2.4. Estimation of $I_{3}$

Let $Y_{1},\ldots ,Y_{n}$ be an independent copy of $X_{1},\ldots ,X_{n}$. We will use the following lemma which is a Bernstein-type exponential inequality for degenerate *U*-statistics.

**Lemma** **1** ((3.5) of Giné, Latała and Zinn [19])**.** *For bounded degenerate kernel $h_{i,j}\left(X_{i},Y_{j}\right)$, let*

$$\begin{array}{c} A=\max_{i,j}{∥h_{i,j}\left(X_{i},Y_{j}\right)∥}_{\infty },\;C^{2}=\sum_{i,j}Eh_{i,j}^{2}\left(X_{i},Y_{j}\right),\;\;\\ B^{2}=\max_{i,j}\left\{∥\sum_{i}Eh_{i,j}^{2}\left(X_{i},y\right){∥}_{\infty },∥\sum_{j}Eh_{i,j}^{2}\left(x,Y_{j}\right){∥}_{\infty }\right\}. \end{array}$$

*Then, there is a universal constant K such that*

$$\begin{array}{c} Pr\left\{|\sum_{i,j}h_{i,j}\left(X_{i},Y_{j}\right)|>x\right\}\leq K\exp\left\{-\frac{1}{K}\min\left[\frac{x}{C},{\left(\frac{x}{B}\right)}^{2/3},{\left(\frac{x}{A}\right)}^{1/2}\right]\right\}. \end{array}$$

Recall (16). Hence, by (19) and the definition of $I_{3}$ in (22),

(36) $$\begin{array}{c} I_{3}\leq P\left(\frac{\sum_{l=m+1}^{\infty }\lambda_{l}{\left({\bar{Y}}_{n,l}-E{\bar{Y}}_{n,l}\right)}^{2}}{\sum_{l=m+1}^{\infty }\lambda_{l}L_{l}\left(z_{n,l}\right)}\geq C_{1}n{\left(1-2\varepsilon \right)}^{2}x_{n}^{2}\right). \end{array}$$

Let

(37) $$\begin{array}{c} h_{m}\left(X_{i},Y_{j}\right)=\sum_{l=m+1}^{\infty }\lambda_{l}\left({\bar{g}}_{l}\left(X_{i}\right)-E{\bar{g}}_{l}\left(X_{i}\right)\right)\left({\bar{g}}_{l}\left(Y_{j}\right)-E{\bar{g}}_{l}\left(Y_{j}\right)\right). \end{array}$$

In addition to the estimate of $I_{3}$, we include (40) in the following proposition which will be used in the proof of Theorem 2, where

(38) $$\begin{array}{ccc} & & h_{\left(\beta \right)}\left(X_{i},Y_{j}\right)\\ & = & \sum_{l=1}^{\infty }\lambda_{l}\left\{(g_{l}\left(X_{i}\right)I(|g_{l}\left(X_{i}\right)|\leq \beta z_{n,l})-Eg_{l}\left(X_{i}\right)I(|g_{l}\left(X_{i}\right)\left|\leq \beta z_{n,l}\right)\right\}\\ & & \times \left\{(g_{l}\left(Y_{j}\right)I(|g_{l}\left(Y_{j}\right)|\leq \beta z_{n,l})-Eg_{l}\left(Y_{j}\right))I(|g_{l}\left(Y_{j}\right)\left|\leq \beta z_{n,l}\right)\right\}. \end{array}$$

**Proposition** **3.** *For a sufficiently large constant $C_{2}>0$,*

(39) $$\begin{array}{c} P\left(\frac{\sum_{1\leq i,j\leq n}h_{m}\left(X_{i},Y_{j}\right)}{n\sum_{l=m+1}^{\infty }\lambda_{l}L_{l}\left(z_{n,l}\right)}\geq C_{2}x_{n}^{2}\right)\leq \exp\left(-3x_{n}^{2}\right), \end{array}$$

*Then, by (36) and the decoupling inequalities of de la Peña and Montgomery-Smith [20], for a sufficiently large $C_{1}>0$,*

$$\begin{array}{c} I_{3}\leq P\left(\frac{\sum_{1\leq i,j\leq n}h_{m}\left(X_{i},X_{j}\right)}{n\sum_{l=m+1}^{\infty }\lambda_{l}L_{l}\left(z_{n,l}\right)}\geq C_{1}{\left(1-2\varepsilon \right)}^{2}x_{n}^{2}\right)\leq \exp\left(-2x_{n}^{2}\right). \end{array}$$

*Suppose that $\lambda_{l}>0$ for all 1≤l<∞ and d>0 is a constant. For constants 0<α,β≤1 that are sufficiently small,*

(40) $$\begin{array}{c} P\left(\frac{\sum_{1\leq i,j\leq [\alpha n]}h_{\left(\beta \right)}\left(X_{i},Y_{j}\right)}{n\sum_{l=1}^{\infty }\lambda_{l}L_{l}\left(z_{n,l}\right)}\geq dx_{n}^{2}\right)\leq \exp\left(-2x_{n}^{2}\right). \end{array}$$

**Proof.**  We will prove (39) and (40) simultaneously. By (15) and (37),

(41) $$\begin{array}{ccc} A_{n} & := & {∥h_{m}\left(X_{i},Y_{j}\right)∥}_{\infty }\leq 4\sum_{l=m+1}^{\infty }\lambda_{l}z_{n,l}^{2}=4\sum_{l=m+1}^{\infty }\lambda_{l}\frac{nL_{l}\left(z_{n,l}\right)}{x_{n}^{2}}. \end{array}$$

By (15) and (38),

(42) $$\begin{array}{c} A_{n,(\beta )}:={∥h_{\left(\beta \right)}\left(X_{i},Y_{j}\right)∥}_{\infty }\leq 4\sum_{l=1}^{\infty }\lambda_{l}\beta^{2}z_{n,l}^{2}=\frac{4\beta^{2}n\sum_{l=1}^{\infty }\lambda_{l}L_{l}\left(z_{n,l}\right)}{x_{n}^{2}}. \end{array}$$

Let

$$B_{n}^{2}:=\max\left\{{∥\sum_{1\leq i\leq n}Eh_{m}^{2}\left(X_{i},y\right)∥}_{\infty },\;{∥\sum_{1\leq j\leq n}Eh_{m}^{2}\left(x,Y_{j}\right)∥}_{\infty }\right\}.$$

Since $|{\bar{g}}_{l}\left(Y_{j}\right)|\leq z_{n,l}$, then by Cauchy–Schwarz inequality, (15) and (37),

$$\begin{array}{ccc} & & {∥\sum_{1\leq i\leq n}Eh_{m}^{2}\left(X_{i},y\right)∥}_{\infty }\leq nE{\left(2\sum_{l=m+1}^{\infty }\lambda_{l}\left|{\bar{g}}_{l}\left(X_{1}\right)-E{\bar{g}}_{l}\left(X_{1}\right)\right|z_{n,l}\right)}^{2}\\ & \leq & 4nE\sum_{l=m+1}^{\infty }\lambda_{l}{\left({\bar{g}}_{l}\left(X_{1}\right)-E{\bar{g}}_{l}\left(X_{1}\right)\right)}^{2}\sum_{l=m+1}^{\infty }\lambda_{l}z_{n,l}^{2}\\ & \leq & 4n\sum_{l=m+1}^{\infty }\lambda_{l}L_{l}\left(z_{n,l}\right)\sum_{l=m+1}^{\infty }\lambda_{l}\frac{nL_{l}\left(z_{n,l}\right)}{x_{n}^{2}}. \end{array}$$

The same result can be obtained for $∥\sum_{1\leq j\leq n}Eh_{m}^{2}\left(x,Y_{j}\right){∥}_{\infty }$. Therefore,

(43) $$\begin{array}{c} B_{n}^{2}\leq \frac{4n^{2}{\left(\sum_{l=m+1}^{\infty }\lambda_{l}L_{l}\left(z_{n,l}\right)\right)}^{2}}{x_{n}^{2}}. \end{array}$$

Similarly,

$$\begin{array}{ccc} B_{n,\alpha ,(\beta )}^{2} & := & \max\left\{{∥\sum_{1\leq i\leq [\alpha n]}Eh_{\left(\beta \right)}^{2}\left(X_{i},y\right)∥}_{\infty },{∥\sum_{1\leq j\leq [\alpha n]}Eh_{\left(\beta \right)}^{2}\left(x,Y_{j}\right)∥}_{\infty }\right\}\\ & \leq & 4\alpha n\sum_{l=1}^{\infty }\lambda_{l}L_{l}\left(\beta z_{n,l}\right)\sum_{l=1}^{\infty }\lambda_{l}\beta^{2}\frac{nL_{l}\left(z_{n,l}\right)}{x_{n}^{2}}. \end{array}$$

Since 0<β≤1, then $L_{l}\left(\beta z_{n,l}\right)/L_{l}\left(z_{n,l}\right)\leq 1$. Hence,

$$\begin{array}{c} B_{n,\alpha ,(\beta )}^{2}\leq \frac{4\alpha \beta^{2}n^{2}{\left(\sum_{l=1}^{\infty }\lambda_{l}L_{l}\left(z_{n,l}\right)\right)}^{2}}{x_{n}^{2}}. \end{array}$$

By (37) and the Cauchy–Schwarz inequality,

(44) $$\begin{array}{ccc} C_{n}^{2} & := & \sum_{1\leq i,j\leq n}Eh_{m}^{2}\left(X_{i},Y_{j}\right)\\ & \leq & \sum_{1\leq i,j\leq n}\sum_{l=m+1}^{\infty }\lambda_{l}E{\left({\bar{g}}_{l}\left(X_{i}\right)-E{\bar{g}}_{l}\left(X_{i}\right)\right)}^{2}\sum_{l=m+1}^{\infty }\lambda_{l}E{\left({\bar{g}}_{l}\left(Y_{j}\right)-E{\bar{g}}_{l}\left(Y_{j}\right)\right)}^{2}\\ & \leq & n^{2}{\left(\sum_{l=m+1}^{\infty }\lambda_{l}L_{l}\left(z_{n,l}\right)\right)}^{2}. \end{array}$$

Similarly,

(45) $$\begin{array}{ccc} C_{n,\alpha ,(\beta )}^{2} & := & \sum_{1\leq i\neq j\leq [\alpha n]}Eh_{\left(\beta \right)}^{2}\left(X_{i},Y_{j}\right)\\ & \leq & \alpha^{2}n^{2}{\left(\sum_{l=1}^{\infty }\lambda_{l}L_{l}\left(\beta z_{n,l}\right)\right)}^{2}\\ & \leq & \alpha^{2}n^{2}{\left(\sum_{l=1}^{\infty }\lambda_{l}L_{l}\left(z_{n,l}\right)\right)}^{2}. \end{array}$$

Now let

(46) $$\begin{array}{c} x=C_{2}nx_{n}^{2}\sum_{l=m+1}^{\infty }\lambda_{l}L_{l}\left(z_{n,l}\right). \end{array}$$

By (41) and (46),

$$\begin{array}{c} {\left(\frac{x}{A_{n}}\right)}^{1/2}\geq {\left(\frac{C_{2}nx_{n}^{2}\sum_{l=m+1}^{\infty }\lambda_{l}L_{l}\left(z_{n,l}\right)}{4n\sum_{l=m+1}^{\infty }\lambda_{l}L_{l}\left(z_{n,l}\right)/x_{n}^{2}}\right)}^{1/2}={\left(C_{2}/4\right)}^{1/2}x_{n}^{2}. \end{array}$$

By (43) and (46),

$$\begin{array}{c} {\left(\frac{x}{B_{n}}\right)}^{2/3}\geq {\left(\frac{C_{2}nx_{n}^{2}\sum_{l=m+1}^{\infty }\lambda_{l}L_{l}\left(z_{n,l}\right)}{2n\sum_{l=m+1}^{\infty }\lambda_{l}L_{l}\left(z_{n,l}\right)/x_{n}}\right)}^{2/3}={\left(C_{2}/2\right)}^{2/3}x_{n}^{2}. \end{array}$$

By (44) and (46),

$$\begin{array}{c} \frac{x}{C_{n}}\geq \frac{C_{2}nx_{n}^{2}\sum_{l=m+1}^{\infty }\lambda_{l}L_{l}\left(z_{n,l}\right)}{n\sum_{l=m+1}^{\infty }\lambda_{l}L_{l}\left(z_{n,l}\right)}=C_{2}x_{n}^{2}. \end{array}$$

Then, (39) follows from Lemma 1 for a sufficiently large $C_{2}$ value. Similarly, let

(47) $$\begin{array}{c} x_{d}=dnx_{n}^{2}\sum_{l=1}^{\infty }\lambda_{l}L_{l}\left(z_{n,l}\right). \end{array}$$

By (42) and (47),

$$\begin{array}{c} {\left(\frac{x_{d}}{A_{n,(\beta )}}\right)}^{1/2}\geq {\left(\frac{dnx_{n}^{2}\sum_{l=1}^{\infty }\lambda_{l}L_{l}\left(z_{n,l}\right)}{4\beta^{2}n\sum_{l=1}^{\infty }\lambda_{l}L_{l}\left(z_{n,l}\right)/x_{n}^{2}}\right)}^{1/2}=\frac{\sqrt{d}}{2\beta }x_{n}^{2}. \end{array}$$

By (44) and (47),

$$\begin{array}{c} {\left(\frac{x_{d}}{B_{n,\alpha ,(\beta )}}\right)}^{2/3}\geq {\left(\frac{dnx_{n}^{2}\sum_{l=1}^{\infty }\lambda_{l}L_{l}\left(z_{n,l}\right)}{2\sqrt{\alpha }\beta n\sum_{l=1}^{\infty }\lambda_{l}L_{l}\left(z_{n,l}\right)/x_{n}}\right)}^{2/3}={\left(\frac{d}{2\sqrt{\alpha }\beta }\right)}^{2/3}x_{n}^{2}. \end{array}$$

By (45) and (47),

$$\begin{array}{c} \frac{x_{d}}{C_{n,\alpha ,(\beta )}}\geq \frac{dnx_{n}^{2}\sum_{l=1}^{\infty }\lambda_{l}L_{l}\left(z_{n,l}\right)}{\alpha n\sum_{l=1}^{\infty }\lambda_{l}L_{l}\left(z_{n,l}\right)}=\frac{d}{\alpha }x_{n}^{2}. \end{array}$$

Therefore, (40) follows from Lemma 1 for α and β values that are sufficiently small. □

### 2.5. Estimation of $I_{4}$

Lemma 2 below follows Corollary 1(b) of Einmahl [21] and Lemma 4.2 of Lin and Liu [22]. However, we add the condition $\sum_{i=1}^{k_{n}}E{∥\xi_{n,i}∥}^{2}\leq b_{n}^{2}$, and our result is in a form of an exponential inequality for independent random vectors. We use the same positive constants $c_{17},c_{20}$ and $c_{22}$ (depending only on the vector dimension *d*) in Einmahl [21].

**Lemma** **2.** *Let $\xi_{n,1},\cdots ,\xi_{n,k_{n}}$ be independent random vectors with a mean of zero and values in $R^{d}$ such that $∥\xi_{n,i}∥\leq A_{n}$ and $\sum_{i=1}^{k_{n}}E{∥\xi_{n,i}∥}^{2}\leq b_{n}^{2}$, where ∥·∥ denotes the Euclidean norm. Let $S_{n}=\sum_{i=1}^{k_{n}}\xi_{n,i}$. Suppose that*

(48) $$\begin{array}{c} Cov\left(S_{n}\right)=B_{n}I_{d} \end{array}$$

*where $B_{n}>0$, $I_{d}$ is a d×d identity matrix, and $\alpha_{n}$ is a positive sequence such that $\alpha_{n}B_{n}^{1/2}\to \infty$ and*

(49) $$\begin{array}{c} \alpha_{n}\sum_{i=1}^{k_{n}}E\left\{∥\xi_{n,i}{∥}^{3}\exp\left(\alpha_{n}∥\xi_{n,i}∥\right)\right\}\leq B_{n}. \end{array}$$

*Let*

(50) $$\begin{array}{c} \beta_{n}=B_{n}^{-3/2}\sum_{i=1}^{k_{n}}E\left\{∥\xi_{n,i}{∥}^{3}\exp\left(\alpha_{n}∥\xi_{n,i}∥\right)\right\}. \end{array}$$

*Then, for any 0<γ<1, there exists $n_{\gamma }$ such that for all $n\geq n_{\gamma }$,*

$$\begin{array}{ccc} P\left(∥S_{n}∥\geq x\right) & \leq & \exp\left\{c_{20}\beta_{n}\left(c_{17}^{3}\alpha_{n}^{3}B_{n}^{3/2}+1\right)\right\}\\ & & \times \left\{\exp\left(-\frac{{\left(1-\gamma \right)}^{6}x^{2}}{2B_{n}}\right)+\exp\left(-\frac{\gamma^{3}{\left(1-\gamma \right)}^{3}x^{2}}{2c_{22}B_{n}\beta_{n}^{2}\log\left(1/\beta_{n}\right)}\right)\right\}\\ & & +2d\exp\left(-\frac{{\left(1-\gamma \right)}^{2}c_{17}^{2}\alpha_{n}^{2}B_{n}^{2}}{2(d^{2}b_{n}^{2}+dc_{17}A_{n}\alpha_{n}B_{n})}\right) \end{array}$$

*uniformly for $x\in [e_{n}B_{n}^{1/2},c_{17}\alpha_{n}B_{n}]$, where ${\left\{e_{n}\right\}}_{n\geq 1}$ can be any sequence with $e_{n}\to \infty$ and $e_{n}\leq c_{17}\alpha_{n}B_{n}^{1/2}$.*

**Proof.**  Let $\eta_{n,i}$, $1\leq i\leq k_{n}$, be independent $N(0,\sigma^{2}Cov\left(\xi_{n,i}\right))$ random vectors, which are independent of the $\xi_{n,i}$s, where

(51) $$\begin{array}{c} \sigma^{2}=c_{22}\beta_{n}^{2}\log\left(1/\beta_{n}\right). \end{array}$$

By (49) and (50), we have $\beta_{n}\leq \alpha_{n}^{-1}B_{n}^{-1/2}\to 0$ as n→∞. Hence, σ→0 as n→∞. Let $p_{n}\left(y\right)$ be the probability density of $B_{n}^{-1/2}\sum_{i=1}^{k_{n}}\left(\xi_{n,i}+\eta_{n,i}\right)$ and $\phi_{\left(1+\sigma^{2}\right)I_{d}}$ be the density of $N(0,\left(1+\sigma^{2}\right)I_{d})$. By Corollary 1(b) in Einmahl [21] (together with the Remark on page 32), for $∥y∥\leq c_{17}\alpha_{n}B_{n}^{1/2}$,

(52) $$\begin{array}{c} p_{n}\left(y\right)=\phi_{\left(1+\sigma^{2}\right)I_{d}}\left(y\right)\exp\left(T_{n}\left(y\right)\right)\;\;\;\mathrm{with}\;\;\;|T_{n}\left(y\right)|\leq c_{20}\beta_{n}{(∥y∥}^{3}+1). \end{array}$$

For any 0<γ<1 and $x\in [e_{n}B_{n}^{1/2},c_{17}\alpha_{n}B_{n}]$,

(53) $$\begin{array}{ccc} & & P\left(∥S_{n}∥\;\geq \;x\right)\\ & \;\leq \; & P\left(∥S_{n}+\sum_{i=1}^{k_{n}}\eta_{n,i}∥\;\geq \;\left(1-\gamma \right)x\right)+P\left(∥\sum_{i=1}^{k_{n}}\eta_{n,i}∥\;\geq \;\gamma x\right)\\ & = & P\left(\left(1-\gamma \right)x\;\leq \;∥S_{n}+\sum_{i=1}^{k_{n}}\eta_{n,i}∥\;<\;c_{17}\alpha_{n}B_{n}\right)\\ & & +P\left(∥S_{n}+\sum_{i=1}^{k_{n}}\eta_{n,i}∥\;\geq \;c_{17}\alpha_{n}B_{n}\right)+P\left(∥\sum_{i=1}^{k_{n}}\eta_{n,i}∥\;\geq \;\gamma x\right)\\ & \;\leq \; & P\left(\left(1-\gamma \right)x\;\leq \;∥S_{n}+\sum_{i=1}^{k_{n}}\eta_{n,i}∥\;<\;c_{17}\alpha_{n}B_{n}\right)\\ & & +P\left(∥S_{n}∥\;\geq \;\left(1-\gamma \right)c_{17}\alpha_{n}B_{n}\right)\\ & & +P\left(∥\sum_{i=1}^{k_{n}}\eta_{n,i}∥\;\geq \;\gamma c_{17}\alpha_{n}B_{n}\right)+P\left(∥\sum_{i=1}^{k_{n}}\eta_{n,i}∥\;\geq \;\gamma x\right)\\ & \;\leq \; & P\left(\left(1-\gamma \right)x\;\leq \;∥S_{n}+\sum_{i=1}^{k_{n}}\eta_{n,i}∥\;<\;c_{17}\alpha_{n}B_{n}\right)+2P\left(∥\sum_{i=1}^{k_{n}}\eta_{n,i}∥\;\geq \;\gamma x\right)\\ & & +P\left(∥S_{n}∥\;\geq \;\left(1-\gamma \right)c_{17}\alpha_{n}B_{n}\right)\\ & := & J_{1}+J_{2}+J_{3}. \end{array}$$

Let *N* denote a centered normal random vector with covariance matrix $I_{d}$. Then, by (52),

(54) $$\begin{array}{ccc} & & J_{1}=\int_{\left(1-\gamma \right)x/B_{n}^{1/2}<∥y∥\leq c_{17}\alpha_{n}B_{n}^{1/2}}\phi_{\left(1+\sigma^{2}\right)I_{d}}\left(y\right)\exp\left(T_{n}\left(y\right)\right)dy\\ & & \leq \exp\left\{c_{20}\beta_{n}\left(c_{17}^{3}\alpha_{n}^{3}B_{n}^{3/2}+1\right)\right\}\int_{∥y∥\geq \left(1-\gamma \right)x/B_{n}^{1/2}}\phi_{\left(1+\sigma^{2}\right)I_{d}}\left(y\right)dy\\ & & \leq \exp\left\{c_{20}\beta_{n}\left(c_{17}^{3}\alpha_{n}^{3}B_{n}^{3/2}+1\right)\right\}\times \\ & & \left\{P\left(∥N∥\geq {\left(1-\gamma \right)}^{2}x/B_{n}^{1/2}\right)+P\left(\sigma ∥N∥\geq \gamma \left(1-\gamma \right)x/B_{n}^{1/2}\right)\right\}. \end{array}$$

Observe that ${∥N∥}^{2}$ has a $\chi_{d}^{2}$ distribution. It is well known that for a $\chi_{d}^{2}$ random variable *Y*, $P\left(Y>y\right)\leq {\left(ye^{1-y/d}/d\right)}^{d/2}$ for y>d. Hence,

(55) $$\begin{array}{cc} \; & P\left(∥N∥\geq {\left(1-\gamma \right)}^{2}x/B_{n}^{1/2}\right)\\ \; & \leq {\left(\frac{{\left(1-\gamma \right)}^{4}x^{2}}{dB_{n}}\exp\left(1-\frac{{\left(1-\gamma \right)}^{4}x^{2}}{dB_{n}}\right)\right)}^{d/2}\\ \; & =\frac{{\left(1-\gamma \right)}^{2d}x^{d}}{d^{d/2}B_{n}^{d/2}}\exp\left(d/2-\frac{{\left(1-\gamma \right)}^{4}x^{2}}{2B_{n}}\right)\\ \; & \leq \exp\left(-\frac{{\left(1-\gamma \right)}^{6}x^{2}}{2B_{n}}\right) \end{array}$$

by $x^{2}/B_{n}\to \infty$. Similarly,

(56) $$\begin{array}{ccc} & & P\left(\sigma ∥N∥\geq \gamma \left(1-\gamma \right)x/B_{n}^{1/2}\right)\leq \exp\left(-\frac{\gamma^{3}{\left(1-\gamma \right)}^{3}x^{2}}{2B_{n}\sigma^{2}}\right)\\ & = & \exp\left(-\frac{\gamma^{3}{\left(1-\gamma \right)}^{3}x^{2}}{2c_{22}B_{n}\beta_{n}^{2}\log\left(1/\beta_{n}\right)}\right) \end{array}$$

by (51). Then, by (54)–(56), we have

(57) $$\begin{array}{ccc} J_{1} & \leq & \exp\left\{c_{20}\beta_{n}\left(c_{17}^{3}\alpha_{n}^{3}B_{n}^{3/2}+1\right)\right\}\\ & & \times \left\{\exp\left(-\frac{{\left(1-\gamma \right)}^{6}x^{2}}{2B_{n}}\right)+\exp\left(-\frac{\gamma^{3}{\left(1-\gamma \right)}^{3}x^{2}}{2c_{22}B_{n}\beta_{n}^{2}\log\left(1/\beta_{n}\right)}\right)\right\}.\;\;\; \end{array}$$

Since the distribution of $\eta_{n,i}$ is $N(0,\sigma^{2}Cov\left(\xi_{n,i}\right))$, the distribution of $\sum_{i=1}^{k_{n}}\eta_{n,i}$ is $N(0,\sigma^{2}\sum_{i=1}^{k_{n}}Cov\left(\xi_{n,i}\right))$. Since the $\xi_{n,i}$s are independent, $\sum_{i=1}^{k_{n}}Cov\left(\xi_{n,i}\right)=Cov\left(\sum_{i=1}^{k_{n}}\xi_{n,i}\right)=B_{n}I_{d}$ by (48). Hence, the distribution of $\sum_{i=1}^{k_{n}}\eta_{n,i}$ is $N(0,\sigma^{2}B_{n}I_{d})$. Then, similar to (56),

(58) $$\begin{array}{ccc} J_{2} & = & 2P\left(∥\sum_{i=1}^{k_{n}}\eta_{n,i}∥\;\geq \gamma x\right)=2P\left(\sigma ∥N∥\geq \gamma x/B_{n}^{1/2}\right)\;\\ & \leq & 2\exp\left(-\frac{\gamma^{3}x^{2}}{2B_{n}c_{22}\beta_{n}^{2}\log\left(1/\beta_{n}\right)}\right). \end{array}$$

By (57) and (58), we have

(59) $$\begin{array}{ccc} & & J_{1}+J_{2}\leq \exp\left\{c_{20}\beta_{n}\left(c_{17}^{3}\alpha_{n}^{3}B_{n}^{3/2}+1\right)\right\}\\ & & \times \left\{\exp\left(-\frac{{\left(1-\gamma \right)}^{6}x^{2}}{2B_{n}}\right)+3\exp\left(-\frac{\gamma^{3}{\left(1-\gamma \right)}^{3}x^{2}}{2c_{22}B_{n}\beta_{n}^{2}\log\left(1/\beta_{n}\right)}\right)\right\}. \end{array}$$

Next, we estimate $J_{3}$. For each 1≤i≤n, let $\xi_{n,i}={\left(\xi_{n,i}^{\left(1\right)},\cdots ,\xi_{n,i}^{\left(d\right)}\right)}^{T}$, where $a^{T}$ denote the transpose of a vector a. Then

$$\begin{array}{c} ∥S_{n}∥=∥\sum_{i=1}^{k_{n}}\xi_{n,i}∥={\left(\sum_{l=1}^{d}{\left(\sum_{i=1}^{k_{n}}\xi_{n,i}^{\left(l\right)}\right)}^{2}\right)}^{1/2}\leq \sum_{l=1}^{d}|\sum_{i=1}^{k_{n}}\xi_{n,i}^{\left(l\right)}|. \end{array}$$

Hence,

$$\begin{array}{ccc} J_{3} & = & P\left(∥S_{n}∥\geq \left(1-\gamma \right)c_{17}\alpha_{n}B_{n}\right)\\ & \leq & P\left(\sum_{l=1}^{d}|\sum_{i=1}^{k_{n}}\xi_{n,i}^{\left(l\right)}|\geq \left(1-\gamma \right)c_{17}\alpha_{n}B_{n}\right)\\ & \leq & \sum_{l=1}^{d}P\left(|\sum_{i=1}^{k_{n}}\xi_{n,i}^{\left(l\right)}|\geq \frac{\left(1-\gamma \right)c_{17}\alpha_{n}B_{n}}{d}\right). \end{array}$$

Since $∥\xi_{n,i}∥\leq A_{n}$ and $\sum_{i=1}^{k_{n}}E{∥\xi_{n,i}∥}^{2}\leq b_{n}^{2}$, then $|\xi_{n,i}^{\left(l\right)}|\leq ∥\xi_{n,i}∥\leq A_{n}$ and $\sum_{i=1}^{k_{n}}E{\left(\xi_{n,i}^{\left(l\right)}\right)}^{2}\leq \sum_{i=1}^{k_{n}}E{∥\xi_{n,i}∥}^{2}\leq b_{n}^{2}$ for each 1≤l≤d. By Bernstein’s inequality (e.g., (2.17) of de la Peña, Lai and Shao [18]),

(60) $$\begin{array}{ccc} J_{3} & \leq & 2d\exp\left(-\frac{{\left(1-\gamma \right)}^{2}c_{17}^{2}\alpha_{n}^{2}B_{n}^{2}}{2d^{2}\left(b_{n}^{2}+c_{17}A_{n}\alpha_{n}B_{n}/d\right)}\right). \end{array}$$

Then, the lemma follows by applying (59) and (60) to (53). □

Now, we estimate $I_{4}$ in the following proposition, which uses some ideas in Liu and Shao [23]:

**Proposition** **4.**
$$\begin{array}{ccc} I_{4} & \leq & P\left(\sum_{l=1}^{m}\lambda_{l}{\left({\bar{Y}}_{n,l}-E{\bar{Y}}_{n,l}\right)}^{2}\geq \left(1-C_{1}\varepsilon \right){\left(1-2\varepsilon \right)}^{3}nx_{n}^{2}\max_{1\leq l\leq m}\lambda_{l}L_{l}\left(z_{n,l}\right)\right)\\ & \leq & \exp\left(-\frac{\left(1-C_{1}\varepsilon \right){\left(1-2\varepsilon \right)}^{4}x_{n}^{2}}{2(1+\varepsilon )}\right). \end{array}$$

**Proof.**  For each 1≤i≤n, let

$$\begin{array}{c} G_{n,i}={\left(\sqrt{\lambda_{1}}\left({\bar{g}}_{1}\left(X_{i}\right)-E{\bar{g}}_{1}\left(X_{i}\right)\right),\cdots ,\sqrt{\lambda_{m}}\left({\bar{g}}_{m}\left(X_{i}\right)-E{\bar{g}}_{m}\left(X_{i}\right)\right)\right)}^{T}. \end{array}$$

Let $B_{n}=n$ and Σ be the covariance matrix of $G_{n,1}$. For 1≤i≤n, let

(61) $$\begin{array}{c} \xi_{n,i}=\Sigma^{-1/2}G_{n,i}. \end{array}$$

Then,

$$\begin{array}{ccc} & & Cov\left(\xi_{n,1}+\cdots +\xi_{n,n}\right)\\ & = & E\left\{\left(\Sigma^{-1/2}\left(G_{n,1}+\cdots +G_{n,n}\right)\right){\left(\Sigma^{-1/2}\left(G_{n,1}+\cdots +G_{n,n}\right)\right)}^{T}\right\}\\ & = & \Sigma^{-1/2}E\left\{{\left(G_{n,1}+\cdots +G_{n,n})(G_{n,1}+\cdots +G_{n,n}\right)}^{T}\right\}\Sigma^{-1/2}\\ & = & \Sigma^{-1/2}\sum_{1\leq i,j\leq n}E\left\{G_{n,i}G_{n,j}^{T}\right\}\Sigma^{-1/2}. \end{array}$$

Since the $X_{i}$s are independent, then

$$\begin{array}{ccc} Cov\left(\xi_{n,1}+\cdots +\xi_{n,n}\right) & = & \Sigma^{-1/2}\sum_{i=1}^{n}E\left\{G_{n,i}G_{n,i}^{T}\right\}\Sigma^{-1/2}\\ & = & nI_{m}=B_{n}I_{m}. \end{array}$$

Hence, Condition (48) in Lemma 2 is satisfied. Let

$$\begin{array}{c} \alpha_{n}=\frac{C_{m}x_{n}}{n^{1/2}} \end{array}$$

where $C_{m}>0$ is a finite constant depending only on *m*. We shall verify Condition (49). By (61),

(62) $$\begin{array}{c} ∥\xi_{n,i}{∥}^{2}={\left(\Sigma^{-1/2}G_{n,i}\right)}^{T}\left(\Sigma^{-1/2}G_{n,i}\right)=G_{n,i}^{T}\Sigma^{-1}G_{n,i}. \end{array}$$

Observe that Σ is positive definite by Assumption (9). Then, by the identity

(63) $$\begin{array}{c} x^{T}A^{-1}x=\max_{∥\vartheta ∥=1}\frac{{\left(x^{T}\vartheta \right)}^{2}}{\vartheta^{T}A\vartheta } \end{array}$$

for any m×m positive definite matrix A, we have

(64) $$\begin{array}{c} ∥\xi_{n,i}{∥}^{2}=G_{n,i}^{T}\Sigma^{-1}G_{n,i}=\max_{∥\vartheta ∥=1}\frac{{\left(G_{n,i}^{T}\vartheta \right)}^{2}}{\vartheta^{T}\Sigma \vartheta }. \end{array}$$

Let $\vartheta^{*}=\left(\vartheta_{1}^{*},\cdots ,\vartheta_{m}^{*}\right)$ such that $∥\vartheta^{*}∥=1$ and ${\left(G_{n,i}^{T}\vartheta^{*}\right)}^{2}=\max_{∥\vartheta ∥=1}{\left(G_{n,i}^{T}\vartheta \right)}^{2}$. Then, for any $\vartheta =\left(\vartheta_{1},\cdots ,\vartheta_{m}\right)\in l^{2}$, by the Cauchy–Schwarz inequality,

(65) $$\begin{array}{ccc} {\left(G_{n,i}^{T}\vartheta \right)}^{2} & = & {\left(\sum_{l=1}^{m}\sqrt{\lambda_{l}}\left({\bar{g}}_{l}\left(X_{i}\right)-E{\bar{g}}_{l}\left(X_{i}\right)\right)\vartheta_{l}\right)}^{2}\\ & = & {\left(\sum_{l=1}^{m}\frac{{\bar{g}}_{l}\left(X_{i}\right)-E{\bar{g}}_{l}\left(X_{i}\right)}{\sqrt{L_{l}\left(z_{n,l}\right)}}\vartheta_{l}\sqrt{\lambda_{l}L_{l}\left(z_{n,l}\right)}\right)}^{2}\\ & \leq & \sum_{l=1}^{m}\frac{{\left({\bar{g}}_{l}\left(X_{i}\right)-E{\bar{g}}_{l}\left(X_{i}\right)\right)}^{2}}{L_{l}\left(z_{n,l}\right)}\sum_{l=1}^{m}\vartheta_{l}^{2}\lambda_{l}L_{l}\left(z_{n,l}\right). \end{array}$$

Since $Eg_{l}\left(X_{1}\right)=0$ for all l≥1, then $E{\bar{g}}_{l}\left(X_{1}\right)=o\left(x_{n}\sqrt{L_{l}\left(z_{n,l}\right)}/\sqrt{n}\right)$ by (13) and (15). By Assumption (9),

$$\begin{array}{ccc} \vartheta^{T}\Sigma \vartheta & = & \sum_{1\leq l,l^{\prime }\leq m}\vartheta_{l}\vartheta_{l^{\prime }}\sqrt{\lambda_{l}\lambda_{l^{\prime }}}E\left({\bar{g}}_{l}\left(X_{1}\right)-E{\bar{g}}_{l}\left(X_{1}\right)\right)\left({\bar{g}}_{l^{\prime }}\left(X_{1}\right)-E{\bar{g}}_{l^{\prime }}\left(X_{1}\right)\right)\\ & = & \sum_{l=1}^{m}\vartheta_{l}^{2}\lambda_{l}E{\bar{g}}_{l}^{2}\left(X_{1}\right)-\sum_{l=1}^{m}\vartheta_{l}^{2}\lambda_{l}{\left(E{\bar{g}}_{l}\left(X_{1}\right)\right)}^{2}\\ & & +\sum_{1\leq l\neq l^{\prime }\leq m}\vartheta_{l}\vartheta_{l^{\prime }}\sqrt{\lambda_{l}\lambda_{l^{\prime }}}E{\bar{g}}_{l}\left(X_{1}\right){\bar{g}}_{l^{\prime }}\left(X_{1}\right)\\ & & -\sum_{1\leq l\neq l^{\prime }\leq m}\vartheta_{l}\vartheta_{l^{\prime }}\sqrt{\lambda_{l}\lambda_{l^{\prime }}}E{\bar{g}}_{l}\left(X_{1}\right)E{\bar{g}}_{l^{\prime }}\left(X_{1}\right)\\ & = & \sum_{l=1}^{m}\vartheta_{l}^{2}\lambda_{l}L_{l}\left(z_{n,l}\right)-\sum_{l=1}^{m}\vartheta_{l}^{2}\lambda_{l}\times o\left(\frac{x_{n}^{2}L_{l}\left(z_{n,l}\right)}{n}\right)\\ & & +o\left(1\right)\sum_{1\leq l\neq l^{\prime }\leq m}\vartheta_{l}\vartheta_{l^{\prime }}\sqrt{\lambda_{l}\lambda_{l^{\prime }}L_{l}\left(z_{n,l}\right)L_{l^{\prime }}\left(z_{n,l^{\prime }}\right)}\\ & & -\sum_{1\leq l\neq l^{\prime }\leq m}\vartheta_{l}\vartheta_{l^{\prime }}\sqrt{\lambda_{l}\lambda_{l^{\prime }}}\times o\left(\frac{x_{n}\sqrt{L_{l}\left(z_{n,l}\right)}}{\sqrt{n}}\right)o\left(\frac{x_{n}\sqrt{L_{l^{\prime }}\left(z_{n,l^{\prime }}\right)}}{\sqrt{n}}\right). \end{array}$$

By the Cauchy–Schwarz inequality,

$$\begin{array}{c} \sum_{1\leq l\neq l^{\prime }\leq m}\vartheta_{l}\vartheta_{l^{\prime }}\sqrt{\lambda_{l}\lambda_{l^{\prime }}L_{l}\left(z_{n,l}\right)L_{l^{\prime }}\left(z_{n,l^{\prime }}\right)}\leq m\sum_{l=1}^{m}\vartheta_{l}^{2}\lambda_{l}L_{l}\left(z_{n,l}\right). \end{array}$$

Hence,

(66) $$\begin{array}{c} \vartheta^{T}\Sigma \vartheta =\left(1+o\left(1\right)\right)\sum_{l=1}^{m}\vartheta_{l}^{2}\lambda_{l}L_{l}\left(z_{n,l}\right). \end{array}$$

Applying (65) and (66) to (64), we have

(67) $$\begin{array}{c} ∥\xi_{n,i}{∥}^{2}\leq 2\sum_{l=1}^{m}\frac{{\left({\bar{g}}_{l}\left(X_{i}\right)-E{\bar{g}}_{l}\left(X_{i}\right)\right)}^{2}}{L_{l}\left(z_{n,l}\right)}. \end{array}$$

Since $|{\bar{g}}_{l}\left(X_{i}\right)|\leq z_{n,l}=\sqrt{nL_{l}\left(z_{n,l}\right)}/x_{n}$ by (15), and (16), we have

(68) $$\begin{array}{c} ∥\xi_{n,i}{∥}^{2}\leq \frac{4mn}{x_{n}^{2}}. \end{array}$$

By (67),

(69) $$\begin{array}{c} E∥\xi_{n,i}{∥}^{2}\leq 2\sum_{l=1}^{m}\frac{E{\left({\bar{g}}_{l}\left(X_{i}\right)-E{\bar{g}}_{l}\left(X_{i}\right)\right)}^{2}}{L_{l}\left(z_{n,l}\right)}\leq 2m \end{array}$$

and

(70) $$\begin{array}{ccc} E∥\xi_{n,i}{∥}^{3} & \leq & 2^{3/2}E{\left(\sum_{l=1}^{m}\frac{{\left({\bar{g}}_{l}\left(X_{i}\right)-E{\bar{g}}_{l}\left(X_{i}\right)\right)}^{2}}{L_{l}\left(z_{n,l}\right)}\right)}^{3/2}. \end{array}$$

By Hölder’s inequality,

(71) $$\begin{array}{c} {\left(\sum_{l=1}^{m}\frac{{\left({\bar{g}}_{l}\left(X_{i}\right)-E{\bar{g}}_{l}\left(X_{i}\right)\right)}^{2}}{L_{l}\left(z_{n,l}\right)}\right)}^{3/2}\leq m^{1/2}\sum_{l=1}^{m}\frac{|{\bar{g}}_{l}\left(X_{i}\right)-E{\bar{g}}_{l}\left(X_{i}\right){|}^{3}}{L_{l}^{3/2}\left(z_{n,l}\right)}. \end{array}$$

Combining (70) and (71), we have

(72) $$\begin{array}{ccc} E∥\xi_{n,i}{∥}^{3} & \leq & 2^{3/2}m^{1/2}\sum_{l=1}^{m}\frac{8E|{\bar{g}}_{l}\left(X_{i}\right){|}^{3}}{L_{l}^{3/2}\left(z_{n,l}\right)}\\ & = & \sum_{l=1}^{m}\frac{o(z_{n,l}L_{l}\left(z_{n,l}\right))}{L_{l}^{3/2}\left(z_{n,l}\right)}=o\left(\frac{n^{1/2}}{x_{n}}\right) \end{array}$$

by (14) and (15). Since $B_{n}=n$ and $\alpha_{n}=C_{m}x_{n}/n^{1/2}$, then by (68) and (72), we have

$$\begin{array}{ccc} & & \alpha_{n}\sum_{i=1}^{n}E\left\{∥\xi_{n,i}{∥}^{3}\exp\left(\alpha_{n}∥\xi_{n,i}∥\right)\right\}\\ & \leq & \frac{C_{m}x_{n}}{n^{1/2}}n\times o\left(\frac{n^{1/2}}{x_{n}}\right)\exp\left(\frac{C_{m}x_{n}}{n^{1/2}}{\left(\frac{4mn}{x_{n}^{2}}\right)}^{1/2}\right)=o\left(n\right)=o\left(B_{n}\right). \end{array}$$

Hence, Condition (49) in Lemma 2 is satisfied. Similarly,

(73) $$\begin{array}{ccc} \beta_{n} & := & B_{n}^{-3/2}\sum_{i=1}^{n}E\left\{∥\xi_{n,i}{∥}^{3}\exp\left(\alpha_{n}∥\xi_{n,i}∥\right)\right\}\\ & = & n^{-3/2}n\times o\left(\frac{n^{1/2}}{x_{n}}\right)\exp{\left(\frac{C_{m}x_{n}}{n^{1/2}}(\frac{4mn}{x_{n}^{2}}\right)}^{1/2})\\ & = & o(1/x_{n}). \end{array}$$

Then, $\beta_{n}^{2}\log\left(1/\beta_{n}\right)=o\left(1/x_{n}\right)$. By (68), we have $∥\xi_{n,i}∥\leq {\left(4mn/x_{n}^{2}\right)}^{1/2}:=A_{n}$. By (69), we have $\sum_{i=1}^{n}E{∥\xi_{n,i}∥}^{2}\leq 2mn:=b_{n}^{2}$. Then, by Lemma 2 and (73) with $B_{n}=n$ and $\alpha_{n}=C_{m}x_{n}/n^{1/2}$ for a sufficiently large $C_{m}$ value, we have

$$\begin{array}{ccc} & & P\left(∥S_{n}∥\geq n^{1/2}x_{n}\right)\\ & \leq & \exp\left\{o(x_{n}^{2})\right\}\left\{\exp\left(-\frac{{\left(1-\gamma \right)}^{6}nx_{n}^{2}}{2n}\right)+\exp\left(-\frac{\gamma^{3}{\left(1-\gamma \right)}^{3}nx_{n}^{2}}{n\times o(1/x_{n})}\right)\right\}\\ & & +2m\exp\left(-\frac{{\left(1-\gamma \right)}^{2}c_{17}^{2}C_{m}^{2}x_{n}^{2}n}{2(2m^{3}n+m{\left(4mn/x_{n}^{2}\right)}^{1/2}c_{17}C_{m}x_{n}n^{1/2})}\right)\\ & \leq & \exp\left\{o(x_{n}^{2})\right\}\left\{\exp\left(-\frac{{\left(1-\gamma \right)}^{6}x_{n}^{2}}{2}\right)+\exp\left(-4x_{n}^{2}\right)\right\}+\exp\left(-4x_{n}^{2}\right)\\ & \leq & \exp\left(-\frac{{\left(1-\gamma \right)}^{7}x_{n}^{2}}{2}\right). \end{array}$$

Letting $\gamma =1-{\left(1-\varepsilon \right)}^{1/7}$, we have

(74) $$\begin{array}{c} P\left(∥S_{n}∥\;\geq n^{1/2}x_{n}\right)\leq \exp\left(-\frac{\left(1-\varepsilon \right)x_{n}^{2}}{2}\right). \end{array}$$

Similar to (62),

(75) $$\begin{array}{ccc} ∥S_{n}{∥}^{2}=∥\sum_{i=1}^{n}\xi_{n,i}{∥}^{2} & = & {\left(\Sigma^{-1/2}\sum_{i=1}^{n}G_{n,i}\right)}^{T}\left(\Sigma^{-1/2}\sum_{i=1}^{n}G_{n,i}\right)\\ & = & {\left(\sum_{i=1}^{n}G_{n,i}\right)}^{T}\Sigma^{-1}\left(\sum_{i=1}^{n}G_{n,i}\right). \end{array}$$

We will use Identity (63) to estimate (75). Let $\vartheta^{*}=\left(\vartheta_{1}^{*},\cdots ,\vartheta_{m}^{*}\right)$ be such that $∥\vartheta^{*}∥\;=1$ and

(76) $$\begin{array}{c} {\left(\sum_{i=1}^{n}G_{n,i}\right)}^{T}\vartheta^{*}=\max_{∥\vartheta ∥=1}{\left(\sum_{i=1}^{n}G_{n,i}\right)}^{T}\vartheta . \end{array}$$

Observe that

(77) $$\begin{array}{c} \max_{∥\vartheta ∥=1}{\left(\sum_{i=1}^{n}G_{n,i}\right)}^{T}\vartheta ={\left(\sum_{l=1}^{m}{\left(\sum_{i=1}^{n}\sqrt{\lambda_{l}}\left({\bar{g}}_{l}\left(X_{i}\right)-E{\bar{g}}_{l}\left(X_{i}\right)\right)\right)}^{2}\right)}^{1/2}. \end{array}$$

By (66),

(78) $$\begin{array}{ccc} {\left(\vartheta^{*}\right)}^{T}\Sigma \vartheta^{*} & = & \left(1+o\left(1\right)\right)\sum_{l=1}^{m}{\left(\vartheta_{l}^{*}\right)}^{2}\lambda_{l}L_{l}\left(z_{n,l}\right)\\ & \leq & \left(1+o\left(1\right)\right)\max_{1\leq l\leq m}\lambda_{l}L_{l}\left(z_{n,l}\right) \end{array}$$

because $∥\vartheta^{*}∥=1$. By Identity (63) and by (76)–(78),

(79) $$\begin{array}{c} {\left(\sum_{i=1}^{n}G_{n,i}\right)}^{T}\Sigma^{-1}\left(\sum_{i=1}^{n}G_{n,i}\right)\geq \frac{\sum_{l=1}^{m}{\left(\sum_{i=1}^{n}\sqrt{\lambda_{l}}\left({\bar{g}}_{l}\left(X_{i}\right)-E{\bar{g}}_{l}\left(X_{i}\right)\right)\right)}^{2}}{\left(1+\varepsilon \right)\max_{1\leq l\leq m}\lambda_{l}L_{l}\left(z_{n,l}\right)}. \end{array}$$

By (74), (75) and (79), with the application of (16) and (19),

$$\begin{array}{ccc} I_{4} & \leq & P\left(\frac{\sum_{l=1}^{m}\lambda_{l}{\left(\sum_{i=1}^{n}\left({\bar{g}}_{l}\left(X_{i}\right)-E{\bar{g}}_{l}\left(X_{i}\right)\right)\right)}^{2}}{\max_{1\leq l\leq m}\lambda_{l}L_{l}\left(z_{n,l}\right)}\geq \left(1-C_{1}\varepsilon \right){\left(1-2\varepsilon \right)}^{3}nx_{n}^{2}\right)\\ & \leq & P\left({\left(\sum_{i=1}^{n}G_{n,i}\right)}^{T}\Sigma^{-1}\left(\sum_{i=1}^{n}G_{n,i}\right)\geq \frac{\left(1-C_{1}\varepsilon \right){\left(1-2\varepsilon \right)}^{3}nx_{n}^{2}}{1+\varepsilon }\right)\\ & = & P\left(∥S_{n}{∥}^{2}\geq \frac{\left(1-C_{1}\varepsilon \right){\left(1-2\varepsilon \right)}^{3}nx_{n}^{2}}{1+\varepsilon }\right)\\ & \leq & \exp\left(-\frac{\left(1-C_{1}\varepsilon \right){\left(1-2\varepsilon \right)}^{4}x_{n}^{2}}{2(1+\varepsilon )}\right). \end{array}$$

□

Since ε is arbitrary, then the upper bound of Theorem 1 follows from (22) and the estimates of $I_{1}$, $I_{2}$, $I_{3}$ and $I_{4}$.

## 3. The Lower Bound of Theorem 1

Let 0<ε<1 be sufficiently small. For 1≤m<∞ that is sufficiently large, by (26), $\max_{1\leq l<\infty }\lambda_{l}V_{n,l}^{2}=\max_{1\leq l\leq m}\lambda_{l}V_{n,l}^{2}$. Together with (17), we have

(80) $$\begin{array}{ccc} & & P(W_{n}\geq \left(1-\varepsilon \right)x_{n}^{2})\\ & = & P\left(\frac{\sum_{l=1}^{\infty }\lambda_{l}\left({\left\{\sum_{i=1}^{n}g_{l}\left(X_{i}\right)\right\}}^{2}-\sum_{i=1}^{n}g_{l}^{2}\left(X_{i}\right)\right)}{\max_{1\leq l<\infty }\lambda_{l}V_{n,l}^{2}}\geq \left(1-\varepsilon \right)x_{n}^{2}\right)\\ & \geq & P\left(\frac{\sum_{l=1}^{m}\lambda_{l}{\left\{\sum_{i=1}^{n}g_{l}\left(X_{i}\right)\right\}}^{2}}{\max_{1\leq l\leq m}\lambda_{l}V_{n,l}^{2}}\geq x_{n}^{2}\right). \end{array}$$

Let ${\tilde{g}}_{l}\left(X_{i}\right)$ be the random variable with distribution which is of the distribution of $g_{l}\left(X_{i}\right)$ conditioned on $|g_{l}\left(X_{i}\right)|\leq z_{n,l}$. Define ${\tilde{Y}}_{n,l}=\sum_{i=1}^{n}{\tilde{g}}_{l}\left(X_{i}\right)$ and ${\tilde{V}}_{n,l}^{2}=\sum_{i=1}^{n}{\tilde{g}}_{l}^{2}\left(X_{i}\right)$. By the definition of $L_{l}\left(x\right)$ and (12),

$$\begin{array}{cc} \; & E{\tilde{g}}_{l}^{2}\left(X_{i}\right)=E{\bar{g}}_{l}^{2}\left(X_{i}\right)/P(|g_{l}\left(X_{i}\right)\left|\leq z_{n,l}\right)\\ \; & =L_{l}\left(z_{n,l}\right)/P(|g_{l}\left(X_{i}\right)\left|\leq z_{n,l}\right)=L_{l}\left(z_{n,l}\right)\left(1+o\left(1\right)\right). \end{array}$$

Notice that (13) implies $E{\tilde{g}}_{l}\left(X_{1}\right)=o\left(L_{l}\left(z_{n,l}\right)/z_{n,l}\right)$. Then, we have

(81) $$\begin{array}{cc} \; & \sigma_{l}^{2}:=E{\left({\tilde{Y}}_{n,l}-E{\tilde{Y}}_{n,l}\right)}^{2}=nE{\left({\tilde{g}}_{l}\left(X_{1}\right)-E{\tilde{g}}_{l}\left(X_{1}\right)\right)}^{2}\\ \; & =nE{\tilde{g}}_{l}^{2}\left(X_{1}\right)\left(1+o\left(1\right)\right)=nL_{l}\left(z_{n,l}\right)\left(1+o\left(1\right)\right) \end{array}$$

and

$$\begin{array}{c} E{\tilde{V}}_{n,l}^{2}=nL_{l}\left(z_{n,l}\right)\left(1+o\left(1\right)\right). \end{array}$$

Then, for 0<δ<1,

(82) $$\begin{array}{ccc} & & P\left(\frac{\sum_{l=1}^{m}\lambda_{l}{\left\{\sum_{i=1}^{n}g_{l}\left(X_{i}\right)\right\}}^{2}}{\max_{1\leq l\leq m}\lambda_{l}V_{n,l}^{2}}\geq x_{n}^{2}\right)\\ & \geq & P\left(\frac{\sum_{l=1}^{m}\lambda_{l}{\left\{\sum_{i=1}^{n}g_{l}\left(X_{i}\right)\right\}}^{2}}{\max_{1\leq l\leq m}\lambda_{l}V_{n,l}^{2}}\geq x_{n}^{2},\max_{1\leq i\leq n}\left|g_{l}\left(X_{i}\right)\right|\leq z_{n,l},,1\leq l\leq m\right)\\ & = & P\left(\frac{\sum_{l=1}^{m}\lambda_{l}{\left\{\sum_{i=1}^{n}{\tilde{g}}_{l}\left(X_{i}\right)\right\}}^{2}}{\max_{1\leq l\leq m}\lambda_{l}{\tilde{V}}_{n,l}^{2}}\geq x_{n}^{2}\right)P\left(\max_{1\leq i\leq n}\left|g_{l}\left(X_{i}\right)\right|\leq z_{n,l},,1\leq l\leq m\right)\\ & \geq & P\left(\frac{\sum_{l=1}^{m}\lambda_{l}{\left\{\sum_{i=1}^{n}{\tilde{g}}_{l}\left(X_{i}\right)\right\}}^{2}}{\max_{1\leq l\leq m}\lambda_{l}{\tilde{V}}_{n,l}^{2}}\geq x_{n}^{2},{\tilde{V}}_{n,l}^{2}\leq \left(1+2\delta \right)\sigma_{l}^{2},1\leq l\leq m\right)\\ & & \times P\left(\max_{1\leq i\leq n}\left|g_{l}\left(X_{i}\right)\right|\leq z_{n,l},,1\leq l\leq m\right)\\ & \geq & P\left(\frac{\sum_{l=1}^{m}\lambda_{l}{\left\{\sum_{i=1}^{n}{\tilde{g}}_{l}\left(X_{i}\right)\right\}}^{2}}{\max_{1\leq l\leq m}\lambda_{l}\sigma_{l}^{2}}\geq \left(1+2\delta \right)x_{n}^{2}\right)\\ & & \times P\left(\max_{1\leq i\leq n}\left|g_{l}\left(X_{i}\right)\right|\leq z_{n,l},,1\leq l\leq m\right)-\sum_{l=1}^{m}P\left({\tilde{V}}_{n,l}^{2}\geq \left(1+2\delta \right)\sigma_{l}^{2}\right). \end{array}$$

Without the loss of generality, assume that $\max_{1\leq l\leq m}\lambda_{l}\sigma_{l}^{2}=\lambda_{1}\sigma_{1}^{2}$. Then,

$$\begin{array}{cc} \; & P\left(\frac{\sum_{l=1}^{m}\lambda_{l}{\left\{\sum_{i=1}^{n}{\tilde{g}}_{l}\left(X_{i}\right)\right\}}^{2}}{\max_{1\leq l\leq m}\lambda_{l}\sigma_{l}^{2}}\geq \left(1+2\delta \right)x_{n}^{2}\right)\\ \; & \geq P\left(\frac{\lambda_{1}{\left\{\sum_{i=1}^{n}{\tilde{g}}_{1}\left(X_{i}\right)\right\}}^{2}}{\lambda_{1}\sigma_{1}^{2}}\geq \left(1+2\delta \right)x_{n}^{2}\right)\\ \; & \geq P\left(\sum_{i=1}^{n}{\tilde{g}}_{1}\left(X_{i}\right)\geq {\left(1+2\delta \right)}^{1/2}\sigma_{1}x_{n}\right). \end{array}$$

Recall (15) and (81). Take $c=1/x_{n}$; thus, we have $|{\tilde{g}}_{1}\left(X_{1}\right)|\leq z_{n,l}=c\sigma_{l}$. Therefore, by Theorem 5.2.2 in Stout [24], for any γ>0, we have

(83) $$\begin{array}{c} P\left(\sum_{i=1}^{n}{\tilde{g}}_{1}\left(X_{i}\right)\geq {\left(1+2\delta \right)}^{1/2}\sigma_{1}x_{n}\right)\geq \exp\left(-\left(x_{n}^{2}/2\right)\left(1+2\delta \right)\left(1+\gamma \right)\right). \end{array}$$

On the other hand,

(84) $$\begin{array}{cc} \; & P\left(\max_{1\leq i\leq n}\left|g_{l}\left(X_{i}\right)\right|\leq z_{n,l},1\leq l\leq m\right)={\left[P\left(|g_{l}\left(X_{1}\right)|\leq z_{n,l},1\leq l\leq m\right)\right]}^{n}\\ \; & ={\left[1-P\left(|g_{l}\left(X_{1}\right)|\geq z_{n,l},\exists 1\leq l\leq m\right)\right]}^{n}\geq {\left[1-\sum_{l=1}^{m}P\left(|g_{l}\left(X_{1}\right)|\geq z_{n,l}\right)\right]}^{n}\\ \; & \geq \exp\left(-2n\sum_{l=1}^{m}P\left(|g_{l}\left(X_{1}\right)|\geq z_{n,l}\right)\right)=\exp\left(-o\left(x_{n}^{2}\right)\right). \end{array}$$

We apply the following exponential inequality (see Lemma 2.1, Csörgő, Lin and Shao [25]; see also Pruitt [26] and Griffin and Kuelbs [1]) for the rest of the proof.

**Lemma** **3.** *Let $\xi ,\xi_{1},\cdots ,\xi_{n}$ be i.i.d. random variables. Then, for any b,v,s>0,*

$$\begin{array}{cc} \; & P\left(\left|\sum_{i=1}^{n}(\xi_{i}I(|\xi_{i}|\leq b)-E\xi_{i}I(|\xi_{i}\left|\leq b)\right)\right|\geq \frac{ve^{v}nE\xi_{i}^{2}I(|\xi_{i}\left|\leq b\right)}{2b}+\frac{sb}{v}\right)\leq 2e^{-s}. \end{array}$$

*By (14),*

(85) $$\begin{array}{c} E{\tilde{g}}_{1}^{4}\left(X_{i}\right)=o\left(z_{n,1}^{2}L_{1}\left(z_{n,1}\right)\right). \end{array}$$

*In Lemma 3, we take $\xi_{i}={\tilde{g}}_{1}^{2}\left(X_{i}\right),s=x_{n}^{2},b=z_{n,1}^{2}$ and v=1/δ. Notice that $sb/v=\delta \sigma_{1}^{2}$ and $\frac{ve^{v}nE\xi_{i}^{2}I(|\xi_{i}\left|\leq b\right)}{2b}=o\left(\sigma_{1}^{2}\right)$ by (81) and (85). Then,*

(86) $$\begin{array}{cc} \; & P\left({\tilde{V}}_{n,1}^{2}\geq \left(1+2\delta \right)\sigma_{1}^{2}\right)=P\left({\tilde{V}}_{n,1}^{2}-E{\tilde{V}}_{n,1}^{2}\geq \left(1+2\delta \right)\sigma_{1}^{2}-E{\tilde{V}}_{n,1}^{2}\right)\\ \; & \leq P\left(\sum_{i=1}^{n}\left({\tilde{g}}_{1}^{2}\left(X_{i}\right)-E{\tilde{g}}_{1}^{2}\left(X_{i}\right)\right)\geq \delta \left(1+\delta \right)\sigma_{1}^{2}\right)\\ \; & \leq 2\exp(-x_{n}^{2}). \end{array}$$

*Combining (80), (82), (83), (84) and (86) and letting λ,δ→0, we have*

$$\begin{array}{c} P\left(W_{n}\geq \left(1-\varepsilon \right)x_{n}^{2}\right)\geq \exp\left(-x_{n}^{2}/2\right). \end{array}$$

## 4. The Upper Bound of Theorem 2

**Lemma** **4** (Lemma 2.3 of Giné, Kwapień, Latała, and Zinn [15])**.** *There exists a universal constant $C_{3}<\infty$ such that for any kernel h and any two sequences of i.i.d. random variables, we have*

$$\begin{array}{c} P\left(\max_{k\leq m,l\leq n}|\sum_{i\leq k,j\leq l}h\left(X_{i},Y_{j}\right)|\geq t\right)\leq C_{3}P\left(|\sum_{i\leq m,j\leq n}h\left(X_{i},Y_{j}\right)|\geq t/C_{3}\right) \end{array}$$

*for all m,n∈N and all t>0.*

**Proposition** **5.** *Under the assumptions of Theorem 1,*

$$\begin{array}{c} \underset{n\to \infty }{lim sup}\frac{\sum_{l=1}^{\infty }\lambda_{l}{\left(\sum_{i=1}^{n}g_{l}\left(X_{i}\right)\right)}^{2}}{\max_{1\leq l<\infty }\lambda_{l}V_{n,l}^{2}\log\log n}\leq 2\;\;a.s. \end{array}$$

*Consequently,*

$$\begin{array}{c} \underset{n\to \infty }{lim sup}\frac{W_{n}}{\log\log n}\leq 2\;\;a.s. \end{array}$$

**Proof.**  Let $x_{n}\to \infty$ as n→∞. Let θ>1 with θ−1 be sufficiently small. For any positive integer k∈(n,θn], via a similar idea as in (19) with 0<η<1,

(87) $$\begin{array}{ccc} & & P\left(\max_{n<k\leq \theta n}\frac{\sum_{l=1}^{\infty }\lambda_{l}{\left(\sum_{i=1}^{k}g_{l}\left(X_{i}\right)\right)}^{2}}{\max_{1\leq l<\infty }\lambda_{l}V_{k,l}^{2}}\geq 2{\left(1+\eta \right)}^{3}x_{n}^{2}\right)\\ & \leq & P\left(\frac{\sum_{l=1}^{\infty }\lambda_{l}{\left(\sum_{i=1}^{n}g_{l}\left(X_{i}\right)\right)}^{2}}{\max_{1\leq l<\infty }\lambda_{l}V_{n,l}^{2}}\geq 2\left(1-\eta \right){\left(1+\eta \right)}^{3}x_{n}^{2}\right)\\ & & +P\left(\max_{n<k\leq \theta n}\frac{\sum_{l=1}^{\infty }\lambda_{l}{\left(\sum_{i=n+1}^{k}g_{l}\left(X_{i}\right)\right)}^{2}}{\max_{1\leq l<\infty }\lambda_{l}V_{k,l}^{2}}\geq \frac{\eta^{2}{\left(1+\eta \right)}^{3}x_{n}^{2}}{2}\right)\\ & := & H_{1}+H_{2}. \end{array}$$

Notice that (10) implies (8). By (17) and the upper bound of Theorem 1,

(88) $$\begin{array}{c} H_{1}\leq \exp\left(-{\left(1-\eta \right)}^{3/2}{\left(1+\eta \right)}^{3}x_{n}^{2}\right). \end{array}$$

Let 0<δ<1 be a sufficiently small constant. By (19),

(89) $$\begin{array}{ccc} & & H_{2}\leq P\left(2m\max_{1\leq l<\infty }\lambda_{l}V_{n,l}^{2}\leq \left(1-\eta \right)n\sum_{1\leq l<\infty }\lambda_{l}L_{l}\left(z_{n,l}\right)\right)\\ & & +P\left(\max_{n<k\leq \theta n}\frac{\sum_{l=1}^{\infty }\lambda_{l}{\left(\sum_{i=n+1}^{k}g_{l}\left(X_{i}\right)I\left(|g_{l}\left(X_{i}\right)|>\delta \eta z_{n,l}\right)\right)}^{2}}{\max_{1\leq l<\infty }\lambda_{l}V_{k,l}^{2}}\geq \frac{\eta^{4}{\left(1+\eta \right)}^{3}x_{n}^{2}}{2}\right)\\ & & +P(\frac{\max_{n<k\leq \theta n}\sum_{l=1}^{\infty }\lambda_{l}{\left(\sum_{i=n+1}^{k}g_{l}\left(X_{i}\right)I(|g_{l}\left(X_{i}\right)\left|\leq \delta \eta z_{n,l}\right)\right)}^{2}}{n\sum_{1\leq l<\infty }\lambda_{l}L_{l}\left(z_{n,l}\right)}\\ & & \geq \frac{\left(1-\eta \right)\left(1-2\eta \right)\eta^{2}{\left(1+\eta \right)}^{3}x_{n}^{2}}{4m})\\ & & :=H_{2,1}+H_{2,2}+H_{2,3}. \end{array}$$

By (24) in Proposition 1,

(90) $$\begin{array}{c} H_{2,1}\leq \exp\left(-2x_{n}^{2}\right). \end{array}$$

By the Cauchy–Schwarz inequality, for each *k*,

(91) $$\begin{array}{ccc} & & \frac{\sum_{l=1}^{\infty }\lambda_{l}{\left\{\sum_{i=n+1}^{k}\left|g_{l}\left(X_{i}\right)\right|I\left(|g_{l}\left(X_{i}\right)|>\delta \eta z_{n,l}\right)\right\}}^{2}}{\max_{1\leq l<\infty }\lambda_{l}V_{k,l}^{2}}\\ & \leq & \frac{\sum_{l=1}^{\infty }\lambda_{l}\sum_{i=n+1}^{k}g_{l}^{2}\left(X_{i}\right)\sum_{i=n+1}^{k}I\left(|g_{l}\left(X_{i}\right)|>\delta \eta z_{n,l}\right)}{\max_{1\leq l<\infty }\lambda_{l}V_{k,l}^{2}}. \end{array}$$

By (17), for some *m* value that is sufficiently large, the sum of the diagonal terms is as follows:

(92) $$\begin{array}{c} \frac{\sum_{l=1}^{\infty }\lambda_{l}\sum_{i=n+1}^{k}g_{l}^{2}\left(X_{i}\right)I\left(|g_{l}\left(X_{i}\right)|>\delta \eta z_{n,l}\right)}{\max_{1\leq l<\infty }\lambda_{l}V_{k,l}^{2}}\leq m. \end{array}$$

By (91) and (92),

(93) $$\begin{array}{ccc} & & H_{2,2}\leq P\left(\max_{n<k\leq \theta n}\frac{\sum_{n+1\leq i\neq j\leq k}\sum_{l=1}^{\infty }\lambda_{l}g_{l}^{2}\left(X_{i}\right)I(|g_{l}\left(X_{j}\right)\left|>\delta \eta z_{n,l}\right)}{\max_{1\leq l<\infty }\lambda_{l}\sum_{i=n+1}^{k}g_{l}^{2}\left(X_{i}\right)}\geq \frac{\eta^{5}{\left(1+\eta \right)}^{3}x_{n}^{2}}{2}\right)\\ & \leq & P\left(\max_{n<k\leq \theta n}\frac{\sum_{n+1\leq i<j\leq k}\sum_{l=1}^{\infty }\lambda_{l}g_{l}^{2}\left(X_{i}\right)I(|g_{l}\left(X_{j}\right)\left|>\delta \eta z_{n,l}\right)}{\max_{1\leq l<\infty }\lambda_{l}\sum_{i=n+1}^{k}g_{l}^{2}\left(X_{i}\right)}\geq \frac{\eta^{5}{\left(1+\eta \right)}^{3}x_{n}^{2}}{4}\right)\\ & + & P\left(\max_{n<k\leq \theta n}\frac{\sum_{n+1\leq j<i\leq k}\sum_{l=1}^{\infty }\lambda_{l}g_{l}^{2}\left(X_{i}\right)I(|g_{l}\left(X_{j}\right)\left|>\delta \eta z_{n,l}\right)}{\max_{1\leq l<\infty }\lambda_{l}\sum_{i=n+1}^{k}g_{l}^{2}\left(X_{i}\right)}\geq \frac{\eta^{5}{\left(1+\eta \right)}^{3}x_{n}^{2}}{4}\right)\\ & \leq & P\left(\max_{n<k\leq \theta n}\sum_{n+2\leq j\leq k}\frac{\sum_{n+1\leq i<j}\sum_{l=1}^{\infty }\lambda_{l}g_{l}^{2}\left(X_{i}\right)I(|g_{l}\left(X_{j}\right)\left|>\delta \eta z_{n,l}\right)}{\max_{1\leq l<\infty }\lambda_{l}\sum_{n+1\leq i<j}g_{l}^{2}\left(X_{i}\right)}\geq \frac{\eta^{5}{\left(1+\eta \right)}^{3}x_{n}^{2}}{4}\right)\\ & + & P\left(\max_{n<k\leq \theta n}\sum_{n+1\leq j<k}\frac{\sum_{j<i\leq k}\sum_{l=1}^{\infty }\lambda_{l}g_{l}^{2}\left(X_{i}\right)I(|g_{l}\left(X_{j}\right)\left|>\delta \eta z_{n,l}\right)}{\max_{1\leq l<\infty }\lambda_{l}\sum_{j<i\leq k}g_{l}^{2}\left(X_{i}\right)}\geq \frac{\eta^{5}{\left(1+\eta \right)}^{3}x_{n}^{2}}{4}\right)\\ & = & H_{2,2,1}+H_{2,2,2}. \end{array}$$

Let

$$\begin{array}{c} \phi_{j}=\frac{\sum_{n+1\leq i<j}\sum_{l=1}^{\infty }\lambda_{l}g_{l}^{2}\left(X_{i}\right)I(|g_{l}\left(X_{j}\right)\left|>\delta \eta z_{n,l}\right)}{\max_{1\leq l<\infty }\lambda_{l}\sum_{n+1\leq i<j}g_{l}^{2}\left(X_{i}\right)}. \end{array}$$

Then, for any constant t>0,

(94) $$\begin{array}{ccc} H_{2,2,1} & \leq & \left(\sum_{n+2\leq j\leq [\theta n]}\frac{\sum_{n+1\leq i<j}\sum_{l=1}^{\infty }\lambda_{l}g_{l}^{2}\left(X_{i}\right)I(|g_{l}\left(X_{j}\right)\left|>\delta \eta z_{n,l}\right)}{\max_{1\leq l<\infty }\lambda_{l}\sum_{n+1\leq i<j}g_{l}^{2}\left(X_{i}\right)}\geq \frac{\eta^{5}{\left(1+\eta \right)}^{3}x_{n}^{2}}{4}\right)\\ & \leq & Ee^{t\sum_{j=n+2}^{\left[\theta n\right]}\phi_{j}}e^{-t\eta^{5}{\left(1+\eta \right)}^{3}x_{n}^{2}/4}. \end{array}$$

Let $E_{j}$ be the expectation of $X_{j}$ for n+2≤j≤[θn]. Then,

(95) $$\begin{array}{c} Ee^{t\sum_{j=n+2}^{\left[\theta n\right]}\phi_{j}}=E\left(e^{t\sum_{j=n+2}^{[\theta n]-1}\phi_{j}}E_{\left[\theta n\right]}e^{t\phi_{\left[\theta n\right]}}\right). \end{array}$$

Since $|e^{s}-1|\leq e^{0\vee s}\left|s\right|$ for any s∈R and $0\leq \phi_{\left[\theta n\right]}\leq m$ for some *m* value that is sufficiently large, then

$$\begin{array}{ccc} \left|E_{\left[\theta n\right]}e^{t\phi_{\left[\theta n\right]}}-1\right| & \leq & e^{mt}tE_{\left[\theta n\right]}\phi_{\left[\theta n\right]}\\ & = & \frac{e^{mt}t\sum_{n+1\leq i<[\theta n]}\sum_{l=1}^{\infty }\lambda_{l}g_{l}^{2}\left(X_{i}\right)P(|g_{l}\left(X_{\left[\theta n\right]}\right)\left|>\delta \eta z_{n,l}\right)}{\max_{1\leq l<\infty }\lambda_{l}\sum_{n+1\leq i<[\theta n]}g_{l}^{2}\left(X_{i}\right)}. \end{array}$$

By (12) and (15), we have $P(|g_{l}\left(X_{\left[\theta n\right]}\right)\left|>\delta \eta z_{n,l}\right)=o\left(x_{n}^{2}/n\right)$. Then, together with (17),

(96) $$\begin{array}{c} E_{\left[\theta n\right]}e^{t\phi_{\left[\theta n\right]}}=1+o\left(x_{n}^{2}/n\right)=e^{o(x_{n}^{2}/n)}. \end{array}$$

Applying (96) to (95), we have

$$\begin{array}{c} Ee^{t\sum_{j=2}^{\left[\theta n\right]}\phi_{j}}=e^{o(x_{n}^{2}/n)}Ee^{t\sum_{j=n+2}^{[\theta n]-1}\phi_{j}}. \end{array}$$

Similarly,

$$\begin{array}{ccc} Ee^{t\sum_{j=n+2}^{[\theta n]-1}\phi_{j}} & = & E(e^{t\sum_{j=n+2}^{[\theta n]-2}\phi_{j}}E_{n-1}e^{t\phi_{[\theta n]-1}})\\ & = & e^{o(x_{n}^{2}/n)}Ee^{t\sum_{j=n+2}^{[\theta n]-2}\phi_{j}}. \end{array}$$

Continue this process from $X_{\left[\theta n\right]}$ to $X_{n+1}$. Thus, we conclude that

(97) $$\begin{array}{c} Ee^{t\sum_{j=n+2}^{\left[\theta n\right]}\phi_{j}}=e^{\left[\left(\theta -1\right)n\right]\times o\left(x_{n}^{2}/n\right)}=e^{o(x_{n}^{2})}. \end{array}$$

Applying (97) to (94) and letting $t=8/(\eta^{5}{\left(1+\eta \right)}^{3})$, we have

(98) $$\begin{array}{c} H_{2,2,1}\leq \exp\left(-2x_{n}^{2}\right). \end{array}$$

To estimate $H_{2,2,2}$, let

$$\begin{array}{c} \psi_{j,k}=\frac{\sum_{j<i\leq k}\sum_{l=1}^{\infty }\lambda_{l}g_{l}^{2}\left(X_{i}\right)I(|g_{l}\left(X_{j}\right)\left|>\delta \eta z_{n,l}\right)}{\max_{1\leq l<\infty }\lambda_{l}\sum_{j<i\leq k}g_{l}^{2}\left(X_{i}\right)}. \end{array}$$

Then, for any constant t>0,

(99) $$\begin{array}{ccc} H_{2,2,2} & \leq & P\left(\sum_{n+1\leq j<[\theta n]}\max_{j<k\leq \theta n}\psi_{j,k}\geq \frac{\eta^{5}{\left(1+\eta \right)}^{3}x_{n}^{2}}{4}\right)\\ & \leq & Ee^{t\sum_{j=n+1}^{[\theta n]-1}\max_{j<k\leq \theta n}\psi_{j,k}}e^{-t\eta^{5}{\left(1+\eta \right)}^{3}x_{n}^{2}/4}. \end{array}$$

Let $E_{j}$ be the expectation of $X_{j}$ for n+1≤j≤[θn]. Note k>j. Then,

(100) $$\begin{array}{ccc} & & Ee^{t\sum_{j=n+1}^{[\theta n]-1}\max_{j<k\leq \theta n}\psi_{j,k}}\\ & & =E(e^{t\sum_{j=n+2}^{[\theta n]-1}\max_{j<k\leq \theta n}\psi_{j,k}}E_{n+1}e^{t\max_{n+1<k\leq \theta n}\psi_{n+1,k}}). \end{array}$$

Observe that

(101) $$\begin{array}{c} \psi_{j,k}=\frac{\sum_{l=1}^{\infty }\lambda_{l}\left(\sum_{j<i\leq k}g_{l}^{2}\left(X_{i}\right)\right)I(|g_{l}\left(X_{j}\right)\left|>\delta \eta z_{n,l}\right)}{\max_{1\leq l<\infty }\lambda_{l}\sum_{j<i\leq k}g_{l}^{2}\left(X_{i}\right)}. \end{array}$$

Then, by (17), $0\leq \psi_{n+1,k}\leq m$ for some *m* value that is sufficiently large. Since $|e^{s}-1|\leq e^{0\vee s}\left|s\right|$ for any s∈R,

(102) $$\begin{array}{ccc} & & \left|E_{n+1}e^{t\max_{n+1<k\leq \theta n}\psi_{n+1,k}}-1\right|\leq e^{mt}tE_{n+1}\max_{n+1<k\leq \theta n}\psi_{n+1,k}. \end{array}$$

Under Assumption (10), for each l∈[1,∞),

$$\begin{array}{c} \lambda_{l}V_{n,l}^{2}=\sum_{i=1}^{n}\lambda_{l}g_{l}^{2}\left(X_{i}\right)\leq c_{l}\sum_{\nu =1}^{\infty }\sum_{i=1}^{n}\lambda_{\nu }g_{\nu }^{2}\left(X_{i}\right)=c_{l}\sum_{\nu =1}^{\infty }\lambda_{\nu }V_{n,\nu }^{2}. \end{array}$$

Recall that (17); then, for each l∈[1,∞),

$$\begin{array}{c} \frac{\lambda_{l}V_{n,l}^{2}}{\max_{1\leq l<\infty }\lambda_{l}V_{n,l}^{2}}\leq \frac{mc_{l}}{1-\varepsilon }. \end{array}$$

Hence, by (101),

$$\begin{array}{c} \psi_{j,k}\leq \frac{m}{1-\varepsilon }\sum_{l=1}^{\infty }c_{l}I(|g_{l}\left(X_{j}\right)\left|>\delta \eta z_{n,l}\right). \end{array}$$

Then,

$$\begin{array}{c} E_{n+1}\max_{n+1<k\leq \theta n}\psi_{n+1,k}\leq \frac{m}{1-\varepsilon }\sum_{l=1}^{\infty }c_{l}P(|g_{l}\left(X_{n+1}\right)\left|>\delta \eta z_{n,l}\right). \end{array}$$

By (12) and (15), we have $P(|g_{l}\left(X_{n+1}\right)\left|>\delta \eta z_{n,l}\right)=o\left(x_{n}^{2}/n\right)$. Then, together with (10),

(103) $$\begin{array}{c} E_{n+1}\max_{n+1<k\leq \theta n}\psi_{n+1,k}=o\left(x_{n}^{2}/n\right). \end{array}$$

Then, by (102) and (103),

$$\begin{array}{c} E_{n+1}e^{t\max_{n+1<k\leq \theta n}\psi_{n+1,k}}=1+o\left(x_{n}^{2}/n\right)=e^{o(x_{n}^{2}/n)}. \end{array}$$

Continue this process from j=n+2 to j=[θn]−1 and by (100),

(104) $$\begin{array}{c} Ee^{t\sum_{j=n+1}^{[\theta n]-1}\max_{j<k\leq \theta n}\psi_{j,k}}=e^{o(x_{n}^{2})}. \end{array}$$

Applying (104) to (99) and letting $t=8/(\eta^{5}{\left(1+\eta \right)}^{3})$, we have

(105) $$\begin{array}{c} H_{2,2,2}\leq \exp\left(-2x_{n}^{2}\right). \end{array}$$

By (93), (98) and (105),

(106) $$\begin{array}{c} H_{2,2}\leq 2\exp\left(-2x_{n}^{2}\right). \end{array}$$

By the definition of $H_{2,3}$ in (89), and by Lemma 4, there is a constant $0<C^{\prime }<\infty$ such that

$$\begin{array}{c} H_{2,3}\leq C^{\prime }P\left(\frac{\sum_{l=1}^{\infty }\lambda_{l}{\left(\sum_{i=n+1}^{\left[\theta n\right]}g_{l}\left(X_{i}\right)I(|g_{l}\left(X_{i}\right)\left|\leq \delta \eta z_{n,l}\right)\right)}^{2}}{n\sum_{l=1}^{\infty }\lambda_{l}L_{l}\left(z_{n,l}\right)}\geq \frac{\left(1-3\eta \right)\eta^{2}x_{n}^{2}}{4mC^{\prime }}\right). \end{array}$$

Similar to (36),

$$\begin{array}{ccc} H_{2,3} & \leq & C^{\prime }P(\frac{\sum_{l=1}^{\infty }\lambda_{l}{\left(\sum_{i=n+1}^{\left[\theta n\right]}\left\{g_{l}\left(X_{i}\right)I(|g_{l}\left(X_{i}\right)|\leq \delta \eta z_{n,l})-Eg_{l}\left(X_{i}\right)I(|g_{l}\left(X_{i}\right)\left|\leq \delta \eta z_{n,l}\right)\right\}\right)}^{2}}{n\sum_{l=1}^{\infty }\lambda_{l}L_{l}\left(z_{n,l}\right)}\\ & & \geq \frac{{\left(1-3\eta \right)}^{2}\eta^{2}x_{n}^{2}}{4mC^{\prime }}). \end{array}$$

By the decoupling version of (40) in Proposition 3,

(107) $$\begin{array}{c} H_{2,3}\leq C^{\prime }\exp\left(-2x_{n}^{2}\right). \end{array}$$

Combining (89), (90), (106) and (107), we have

(108) $$\begin{array}{c} H_{2}\leq \left(3+C^{\prime }\right)\exp\left(-2x_{n}^{2}\right). \end{array}$$

By (87), (88) and (108),

$$\begin{array}{ccc} & & P\left(\max_{n<k\leq \theta n}\frac{\sum_{l=1}^{\infty }\lambda_{l}{\left(\sum_{i=1}^{k}g_{l}\left(X_{i}\right)\right)}^{2}}{\max_{1\leq l<\infty }\lambda_{l}V_{k,l}^{2}}\geq 2{\left(1+\eta \right)}^{3}x_{n}^{2}\right)\\ & \leq & \exp\left(-{\left(1-\eta \right)}^{7/4}{\left(1+\eta \right)}^{3}x_{n}^{2}\right). \end{array}$$

Let $n=[\theta^{j}]$ for some j∈N. We have

$$\begin{array}{ccc} & & P\left(\max_{\theta^{j}<k\leq \theta^{j+1}}\frac{\sum_{l=1}^{\infty }\lambda_{l}{\left(\sum_{i=1}^{k}g_{l}\left(X_{i}\right)\right)}^{2}}{\max_{1\leq l<\infty }\lambda_{l}V_{k,l}^{2}}\geq 2{\left(1+\eta \right)}^{3}x_{\left[\theta^{j}\right]}^{2}\right)\\ & & \leq \exp\left(-{\left(1-\eta \right)}^{7/4}{\left(1+\eta \right)}^{3}x_{\left[\theta^{j}\right]}^{2}\right). \end{array}$$

Let $x_{n}^{2}=\log\log n$. Then,

$$\begin{array}{ccc} & & \sum_{j=1}^{\infty }P\left(\max_{\theta^{j}<k\leq \theta^{j+1}}\frac{\sum_{l=1}^{\infty }\lambda_{l}{\left(\sum_{i=1}^{k}g_{l}\left(X_{i}\right)\right)}^{2}}{\max_{1\leq l<\infty }\lambda_{l}V_{k,l}^{2}\log\log k}\geq 2{\left(1+\eta \right)}^{3}\right)\\ & \leq & \sum_{j=1}^{\infty }P\left(\max_{\theta^{j}<k\leq \theta^{j+1}}\frac{\sum_{l=1}^{\infty }\lambda_{l}{\left(\sum_{i=1}^{k}g_{l}\left(X_{i}\right)\right)}^{2}}{\max_{1\leq l<\infty }\lambda_{l}V_{k,l}^{2}}\geq 2{\left(1+\eta \right)}^{3}x_{\left[\theta^{j}\right]}^{2}\right)\\ & \leq & \sum_{j=1}^{\infty }\exp\left(-{\left(1-\eta \right)}^{7/4}{\left(1+\eta \right)}^{3}\log\log\left[\theta^{j}\right]\right)\\ & \leq & K\sum_{j=1}^{\infty }\exp\left(-{\left(1-\eta \right)}^{2}{\left(1+\eta \right)}^{3}\log j\right)<\infty . \end{array}$$

By the Borel–Cantelli lemma,

$$\begin{array}{ccc} & & \underset{n\to \infty }{lim sup}\frac{\sum_{l=1}^{\infty }\lambda_{l}{\left(\sum_{i=1}^{n}g_{l}\left(X_{i}\right)\right)}^{2}}{\max_{1\leq l<\infty }\lambda_{l}V_{n,l}^{2}\log\log n}\leq \;2\;\;a.s. \end{array}$$

□

## 5. The Lower Bound of Theorem 2

**Proof.**  By the definition of $W_{n}$,

$$\begin{array}{ccc} \frac{W_{n}}{\log\log n} & = & \frac{\sum_{l=1}^{\infty }\lambda_{l}\left({\left(\sum_{i=1}^{n}g_{l}\left(X_{i}\right)\right)}^{2}-\sum_{i=1}^{n}g_{l}^{2}\left(X_{i}\right)\right)}{\max_{1\leq l<\infty }\lambda_{l}V_{n,l}^{2}\log\log n}\\ & = & \frac{\sum_{l=1}^{\infty }\lambda_{l}{\left(\sum_{i=1}^{n}g_{l}\left(X_{i}\right)\right)}^{2}}{\max_{1\leq l<\infty }\lambda_{l}V_{n,l}^{2}\log\log n}-\frac{\sum_{l=1}^{\infty }\lambda_{l}V_{n,l}^{2}}{\max_{1\leq l<\infty }\lambda_{l}V_{n,l}^{2}\log\log n}. \end{array}$$

By (17), $\sum_{l=1}^{\infty }\lambda_{l}V_{n,l}^{2}/\left(\max_{1\leq l<\infty }\lambda_{l}V_{n,l}^{2}\log\log n\right)\leq m/\left(\left(1-\varepsilon \right)\log\log n\right)\to 0$ as n→∞, we have

$$\begin{array}{c} \frac{W_{n}}{\log\log n}\geq \frac{\sum_{l=1}^{\infty }\lambda_{l}{\left(\sum_{i=1}^{n}g_{l}\left(X_{i}\right)\right)}^{2}}{\max_{1\leq l<\infty }\lambda_{l}V_{n,l}^{2}\log\log n}. \end{array}$$

Then,

$$\begin{array}{c} \frac{W_{n}}{\log\log n}\geq \sum_{k=1}^{\infty }\frac{{\left(\sum_{i=1}^{n}g_{k}\left(X_{i}\right)\right)}^{2}}{V_{n,k}^{2}\log\log n}I_{k=min\{j:\max_{1\leq l<\infty }\lambda_{l}V_{n,l}^{2}=\lambda_{j}V_{n,j}^{2}\}}. \end{array}$$

Hence, by (6),

$$\begin{array}{c} \underset{n\to \infty }{lim sup}\frac{W_{n}}{\log\log n}\geq 2\;\;a.s. \end{array}$$

□

## Data Availability

Data sharing is not applicable (only appropriate if no new data is generated or the article describes entirely theoretical research).
